# Integrative Analyses of m6A Regulators Identify that METTL3 is Associated with HPV Status and Immunosuppressive Microenvironment in HPV-related Cancers

**DOI:** 10.7150/ijbs.70674

**Published:** 2022-06-03

**Authors:** Ruidi Yu, Ye Wei, Chao He, Ping Zhou, Hong Yang, Chang Deng, Rang Liu, Peng Wu, Qinglei Gao, Canhui Cao

**Affiliations:** 1 Department of Gynecology and Obstetrics, Cancer Biology Research Center (Key Laboratory of the Ministry of Education), Tongji Hospital, Tongji Medical College, Huazhong University of Science and Technology, Wuhan, Hubei, China.; 2 Center for Reproductive Medicine, Department of Obstetrics and Gynecology, Peking University Shenzhen Hospital, Shenzhen Peking University-The Hong Kong University of Science and Technology Medical Center, Guangdong, 518036, China.; 3 Center for Reproductive Medicine, Department of Obstetrics and Gynecology, Peking University Third Hospital, Beijing, 100191, China.

**Keywords:** m6A, HPV, tumor immune microenvironment, HNSC, CESC, METTL3

## Abstract

Although m6A modifications are associated with tumor progression, and anti-tumor immune responses, the role of m6A regulators in HPV-related carcinogenesis has not been well resolved. To provide evidence for the role of m6A regulators in HPV-related carcinogenesis and identify potential therapeutic targets for HPV-related cancers, integrative analyses of m6A regulators in 1,485 head and neck squamous cell carcinoma (HNSC) patients and 507 cervical squamous cell carcinoma (CESC) patients was performed and identified that an m6A regulator, METTL3, was highly expressed in tumors and was related to the poor prognosis in HNSC and CESC. In HPV-positive tumors, METTL3 was positively associated with tumor HPV status, such as HPV integration status, E6 and unspliced-E6 expression, and p16 expression. Further analysis demonstrated that METTL3 ^high^ status was negatively correlated with tumor immune cell infiltrations and facilitated the expression of immunosuppressive immune checkpoint molecules (i.e., PD-L1). Cell-derived xenograft models demonstrated that METTL3 inhibitor combined with anti-PD1 therapy promoted immunotherapy of CESC *in vivo*. Overall, this study identified that METTL3^ high^ status, is associated with poor prognosis and HPV status, and serves as a mediator of the immunosuppressive tumor microenvironment in HPV-associated cancer, which provides a promising therapeutic target for anti-cancer immunotherapy.

## Introduction

Human papillomavirus (HPV) was responsible for more than 90% of anal and cervical cancers, about 70% of vaginal and vulvar cancers, 60% of penile cancers, and 60% to 70% of cancers of the oropharynx 1-3. High-risk HPV infections last longer than low-risk HPV infections, which cause about 5% of all cancers worldwide 4, 5. Persistent high-risk HPV infections were thought to lead to genome integration and caused genomic instability, genomic alteration accumulation, and local immune suppression 6.

Several mechanisms have been identified to be involved in the development and progression of HNSC, such as genetic and epigenetic alterations, dysregulation of cellular signaling pathways, and immune evasion 7. Although numerous efforts are made for cervical cancer and HNSC treatment strategies, the prognosis and postoperative survival for cervical cancer patients are still dismal, and their pathogenesis molecular mechanisms and new therapeutic targets are essential 8, 9.

Epigenetics has become a hot topic in scientific research nowadays 10-12. N6-methyladenosine (m6A), a reversible methylation modification at the sixth N atom of adenine, is the most prevalent, abundant, and conserved internal posttranscriptional modification in eukaryotic RNAs 13. It regulates RNA transcript, splicing, processing, translation, and decay, and plays crucial roles in a variety of biological processes 14, 15. m6A modification can be installed by m6A methyltransferases, or writers, removed by demethylases, or erasers, and recognized by m6A-binding proteins, or readers 13. The m6A regulator, which plays a vital regulatory role in immune response, stem cell differentiation, embryonic development, microRNA editing, tumor progression, prognosis, and radiation resistance, determines m6A methylation levels 12, 15-18. Although m6A has been the focus of many studies in recent years, our understanding of it is far from comprehensive, and the potential mechanisms of m6A modification in cancer should be further investigated 12, 15, 17, 18.

In terms of HPV and m6A modification, studies have shown that circE7 possesses m6A modifications in the cytoplasm, and is translated to produce E7, an oncoprotein, yielding novel insights into how HPV regulates infection and tumorigenesis 19. However, to date, few studies have described the relationship between HPV and m6A methylation 20. Recent studies indicated that m6A modification has a close relationship with tumor immune microenvironment (TIME) remodeling, which could affect the development of tumors 21. High m6A modification linked with a non-inflamed and immune-exclusion tumor microenvironment phenotype that lacked effective immune infiltration and exhibited a poorer survival rate for stroma 21, 22. The m6A regulators such as ALKBH5 and YTHDF1 had a close relation to the immune microenvironment remodeling of gliomas 23. Besides, research suggested that the deletion of m6A demethylase ALKBH5 could sensitize tumors to cancer immunotherapy and adjust the composition of tumor-infiltrating Treg cells and myeloid-derived suppressor cells 24

Breaking immune tolerance may be a powerful strategy to overcome immunosuppression and may provide durable responses in a certain percentage of patients with recurrent disease 25. In HNSC, which is characterized by immunosuppression, immunotherapy is a very promising area as it shows more specificity and less toxicity than conventional therapies 26-28. In cervical cancer, immunotherapies are being incorporated into treatment modalities and clinical trials 29. These therapies have the potential to reduce the burden of disease associated with HPV infection and improve the quality of life of patients 29. However, the role and mechanism of m6A in the tumor immune response in HPV-associated cancers are not well defined 26, 30-32.

Here, we compared the mutational characteristics of m6A regulators in CESC and HNSC to provide a mutational profile of m6A regulators. Subsequently, we investigated m6A regulator expression patterns, prognostic relevance, and correlation with HPV status and found that METTL3 was associated with HPV status and poor prognosis in HNSC and CESC. In addition, we explored the immune relevance of METTL3 through cell line experiments, paired specimens from patients, and TCGA data. We aim to provide a promising therapeutic target for anti-cancer immunotherapy in HNSC and CESC.

## Material and Methods

### Cell culture

SiHa, C33A, U14, TC-1, and Caski purchased from ATCC were cultured in high glucose DMEM (12430054, Gibco), supplemented by 10 % fetal bovine serum (SH30406.05, Hyclone) and penicillin-streptomycin (100 U/mL, 15140163, Gibco). Cells were incubated at 37 °C with 5% CO_2_.

### Multispectral fluorescent immunohistochemistry and analysis

Formalin-fixed, paraffin-embedded tissues in 4 µm thick were processed. In brief, slides were heated at 70 °C for 3h; then residual paraffin was removed, and tissue was rehydrated. Antigen retrieval was performed in Tris-EDTA buffer. Then, slides were washed and blocked. Primary antibodies were incubated for 1h in a humidified chamber at room temperature, followed by detection using Goat Anti-Rabbit IgG H&L (HRP). Visualization was accomplished using TSA kits, after which the slide was placed in Tris-EDTA buffer and heated. Serially, the slide was then incubated with the next primary antibodies. After the stain of the last target, nuclei were subsequently visualized with DAPI (Servicebio, G1012-100ML), and the section was coverslipped using Anti Fluorescence Quenching Mounting Agent (Servicebio, G1401-5ML). Multiplexed and single-color control slides were loaded onto the PerkinElmer Vectra automated multispectral microscope. And tissues and cells were segmented and scored.

### Flow cytometry assays

In brief, cells were collected, washed, and blocked. Then, incubate the cells with antibodies at 4 °C for 30 minutes. All antibodies were purchased already conjugated with fluorescent dyes except the anti-human BTN2A2 antibody. A PercP-conjugated goat anti-mouse IgG secondary antibody (Biolegend) was used for BTN2A2 detection. Corresponding isotype control antibody was used for each antibody in all experiments. Data were acquired on a FACS Calibur flow cytometer (BD Biosciences) and analyzed with Flowjo software. Cells were first gated based on forward and side scatters to exclude debris. Mean fluorescence was calculated. The experiments were repeated three times.

### Immunohistochemistry

Tissues were formaldehyde-fixed and paraffin-embedded (FFPE). The 4 μm FFPE sections were deparaffinized and antigen retrieved. Endogenous peroxidase was inactivated by 3% H2O2 to prevent false-positive staining. The non-specific binding proteins were blocked by bovine serum albumin (BSA, 5%, Servicebio). Primary antibody against METTL3 (abcam, ab195352) and p16 (Genetex, GTX03119) was incubated at 4°C overnight, followed by an HRP-linked secondary antibody for 30 min. The slides were then stained with the DAB kit (Servicebio). The score of results was evaluated via Image-Pro Plus.

### Hematoxylin-eosin staining

The formalin-fixed and paraffin-embedded specimens were stained with Hematoxylin-eosin as previously described 33. Briefly, the slides were de-paraffinized and hydrated. Then, they were stained with hematoxylin, differentiated, and blued. Counterstaining was performed using 1% eosin Y followed by dehydration in increasing concentrations of alcohol. The slides were finally cleared in xylene.

### RT-qPCR

Total RNA was routinely extracted using TRIzol reagent (Invitrogen; Thermo Fisher Scientific, Inc.), and RNA quality was examined by Nanodrop (Thermo, Waltham, MA, USA) analysis and gel electrophoresis. The relative quantity of mRNA was determined by RT-qPCR using a CFX96 Touch Real-Time PCR Detection System (Bio-Rad Laboratories, Inc.) with SYBR Green Supermix (Bio-Rad Laboratories, Inc.). The expression levels of genes were quantified using the comparative CT method. The expression level of each mRNA was normalized to the level of GAPDH mRNA and expressed as the fold difference relative to the control. The primer sequences used in RT-qPCR are shown in Supplementary Table 1.

### Western blot

Cells were collected and washed with PBS and then lysed with RIPA lysis buffer (Beyotime) supplemented with a protease inhibitor cocktail (Roche). Total protein amount was measured, and 30 µg total lysate per sample was subjected to SDS-PAGE followed by immunodetection with the following primary antibodies: PD-L1 (CST, 13684T), METTL3 (abcam, ab195352), p16 (Genetex, GTX03119), p21 (CST, 2947T), HPV16 E6/ HPV18 E6 (Santa Cruz Biotechnology, sc-460), and GAPDH (abclonal, A19056). The corresponding HRP-linked secondary antibody (abcam, ab6721) and enhanced chemiluminescence (Pierce) were added.

### Interference of METTL3 expression

The expression of METTL3 in cell lines interfered with siRNA (Ribobio). siRNA was transfected using Lipofectamine 3000 (Thermo Fisher Scientific) according to the protocols on the user guide. In brief, cells were seeded to be 70%-90% confluent at transfection. Subsequently, Lipofectamine 3000 was diluted in Opti-MEM medium (Gibco). The siRNA was mixed with Opti-MEM. Following that, mix the siRNA and Lipofectamine 3000 and incubate for 10-15 minutes at room temperature. Then, discard the medium in the plate and add the siRNA-lipid complex to cells. After 72 hours of incubation at 37 °C, the cells were harvested for the following analysis.

### METTL3 overexpression in cell lines

The plasmids were transfected using X-tremeGENE HP DNA Transfection Reagent. Briefly, plasmid DNA was diluted with serum-free medium to 0.01 μg/μl. Then, we pipetted the X-tremeGENE HP DNA Transfection Reagent into the diluent. The mixture was incubated for 15 minutes at room temperature. Afterward, the transfection compositions were added to the cells in a dropwise manner. Following transfection, incubate cells for 48-72 hours before measuring expression.

### Sample data acquisition

Mutations of 38 m6A regulators were studied in 1,485 HNSC patients and 507 CESC patients through cBioPortal (https://www.cbioportal.org/). Integrated expression profile and clinical characteristics including HPV status were obtained from TCGA dataset. Mutation characteristics of m6A regulators in HNSC and CESC were explored with cBioPortal. A total of 38 common m6A regulators, including writers, readers, and erasers, were included in the analysis. COSMIC Cancer Browser was used to analyze the mutation features in HNSC (Tissue selection: Upper aerodigestive tract (5888/21952)-Sub-tissue selection: Head neck (2166)-Histology selection: Carcinoma (2162)-Sub-histology selection: Squamous cell carcinoma (2116)) and CESC (Tissue selection: Cervix (1076/9899)-Sub-tissue selection: Include all (1076)-Histology selection: Carcinoma (1035)-Sub-histology selection: Squamous cell carcinoma (647)) on July 10^th^, 2021.

### Immune cell infiltration levels evaluation

The relationship between immune infiltration and gene expression was explored using TCGA data with TIMER 2.0. Lollipop plots and scatter plots were shown using the GSVA R package and statistical significance was calculated using Spearman correlation analysis.

### Immune checkpoint correlation analysis

The association between immune checkpoint molecules and gene expression was explored using TCGA data with TIMER 2.0. Scatter plots were drawn using the ggplot2 R package. Spearman correlation analysis was used to calculate statistical differences and correlations.

### Survival analysis

Survival analysis was performed with GEPIA 2. Based on the expression status of the m6A regulators, the overall survival and disease-free survival were analyzed. And the survival map and Kaplan-Meier curve were plotted. Samples were clustered into two groups using the median of the expression level as the cutoff value. The hazards ratio was calculated based on Cox PH model. The log-rank test was used to assess statistical significance.

### Enrichment analysis

The samples were divided into two groups, high and low expression, according to the expression level of METTL3 (METTL3 ^high^, METTL3 ^low^). The DESeq2 (v1.26.0) R package was used to calculate the differentially expressed genes in the two groups. Gene ontology (GO) and Kyoto Encyclopedia of Genes and Genomes (KEGG) analyses of differentially expressed genes were performed using GOplot, org.Hs.eg.db and clusterProfiler packages and visualized using the ggplot2 package.

### Animal experiments

Female BALB/c nude mice (4 weeks old) and female C57BL/6 mice (4 weeks old) were purchased from Jiangsu GemPharmatech Co. Ltd (Jiangsu, China). The mice were maintained in an accredited animal facility at Tongji Hospital. Animal experiments were approved by the Animal Ethics Committee of Tongji Hospital. Animal experiments complied with the ARRIVE guidelines and were carried out in accordance with the U.K. Animals (Scientific Procedures) Act, 1986 and associated guidelines, EU Directive 2010/63/EU for animal experiments, or the National Research Council's Guide for the Care and Use of Laboratory Animals. Manipulators were blinded to the group information. For BALB/c nude mice, the subcutaneous tumor model of cervical cancer cell lines was established as follows. Briefly, mice were randomly assigned to groups. 4.0×10^6^ cells in the mixture of serum-free medium and Matrigel (354230, BD) within 100μL were injected into subcutaneous tissue. The mice were killed 7 weeks after inoculation with tumor cells and their tumors were excised.

For C57BL/6 mice, the subcutaneous tumor model of U14 was established as above. Seven days after injection, mice were randomly assigned to four groups to receive either 50 mg/kg STM2457 (once daily for 14 days), 250 µg/kg monoclonal PD-1 antibody (once every two days for seven times), PBS (once every two days for seven times) or a combination of the first two treatment regimens by intraperitoneal injection.

### Mouse plasma biochemical assay

Blood was collected from the orbits of mice, with heparin as an anticoagulant, and the specimens were centrifuged at 3000 rpm at 4 °C for 15 min within 30 min after collection, and the supernatant was taken for the next step plasma biochemical assay. Alanine transaminase (ALT), aspartate aminotransferase (AST), gamma-glutamyl transferase (γ-GT), total bilirubin (TBIL), lactate dehydrogenase (LDH), UREA, blood urea nitrogen (BUN), and creatinine concentration (CREA) was tested with Rayto Biochemistry analyzer Chemray 420 and reagents (Rayto Life and Analytical Sciences Co., Ltd.).

### Statistical Analysis

Data were presented as mean ± standard deviation. All statistical analyses were performed on the statistical package of GraphPad Prism 9. Survival analysis was expressed by the Kaplan-Meier curve, tested by the Log-Rank test. Spearman correlation analysis was used to assess the correlation between the two molecules and between immune cell infiltration and gene expression. Two experimental groups were compared by using Student's t-test for unpaired data. Where more than two groups were compared, a one-way ANOVA with Bonferroni's correction was used. P < 0.05 was considered significant. Chi-Square test was used when the variable in question is categorical. P values are indicated as *, P < 0.05; **, P < 0.01; ***, P < 0.001; and ****, P < 0.0001.

## Results

### Expression pattern of m6A regulators in HNSC and CESC and clinical relevance

**Figure 1:**
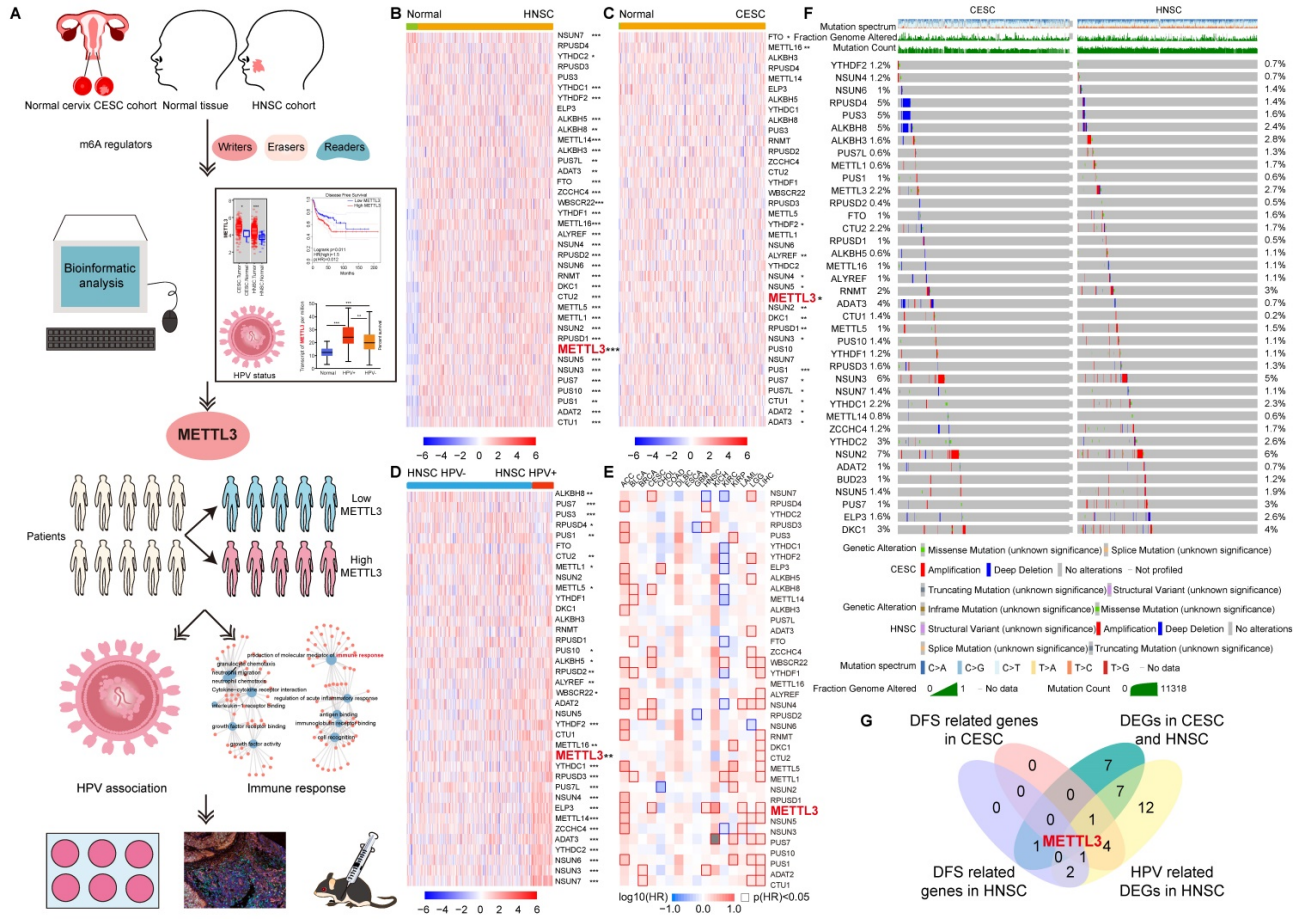
** Expression and variation pattern of m6A regulators in HNSC and CESC and clinical relevance.** (A) Flow chart of the study. Bioinformatics analysis was performed using TCGA data from HNSC and CESC to screen for METTL3, classify patients into METTL3 ^high^ or METTL3 ^low^ status based on their expression, and perform enrichment analysis as well as *in vitro* and *in vivo* experiments for HPV correlation and immune response. (B)The expression spectrum of m6A regulators in HNSC and normal tissue. (C) The expression spectrum of m6A regulators in CESC and normal tissue. (D) The expression spectrum of m6A regulators in HPV positive and negative HNSC. (E) The DFS survival map of m6A regulators. (F) The mutation spectrum of m6A regulators. (G) The Venn of DEGs in HNSC and CESC, HPV-related DEGs in HNSC, and DFS related genes in HNSC and CESC, and METTL3 was screened out. P values are indicated as *, P < 0.05; **, P < 0.01; ***, P < 0.001; and ****, P < 0.0001.

Data were collected from TCGA for 304 CESC samples and 3 corresponding normal tissues and 520 HNSC samples and 44 corresponding normal tissues. We explored the expression profile of 38 m6A regulators in HNSC and CESC using TCGA data (Figure 1A). There were 34 differentially expressed genes (DEGs) identified in HNSC and 17 DEGs identified in CESC (Figure 1B and 1C). The number of overlapping DEGs in HNSC and CESC was 17 (Figure S1A). The expression levels of these 17 overlapping DEGs were shown in Figure S1B. The expression of NUSN3, METTL3, PUS1, PUS7, ALYREF, YTHDF2, ADAT3, NSUN4, PUS7L, NSUN5, NSUN2, RPUSD1, DKC1, ADAT2, and CTU1 was higher in tumors than in normal tissues in both HNSC and CESC. Exceptionally, in HNSC, METTL16 and FTO expression were lower in tumors than in normal tissues and higher in CESC. The DEGs and their expression trends in HNSC and CESC on chromosomes were shown in Figure S1C and S1D. There were more over-expressed genes in HNSC than in CESC. For the differentially expressed m6A regulators, most of them were over-expressed in HNSC and CESC compared to normal control tissues (Figure S1C and S1D).

To investigate the m6A regulators associated with HPV, we studied the expression of 38 m6A regulators in 148 (143 HPV-positive and 5 HPV-negative) CESC samples and 490 (72 HPV-positive and 418 HPV-negative) HNSC samples (Figure 1D and Figure S2). There were 28 genes whose expressions were related to HPV positive or negative in HNSC (Figure S2). Except for PUS7, PUS1, METTL1, ALKBH8, PUS3, RPUSD4, METTL5, and CTU2, other HPV-associated m6a regulators expressed more in HPV-positive HNSC than in HPV-negative HNSC. Taking the intersection of DEGs in CESC, DEGs in HNSC, and HPV-related m6A regulators in HNSC, 10 HPV-related m6A regulators were identified, namely NUSN3, METTL3, PUS1, PUS7, ALYREF, YTHDF2, ADAT3, NSUN4, PUS7L, and METTL16 (Figure S3A and S3B).

The survival contribution of m6A regulator genes in multiple cancer types was shown in the survival map (Figure 1E and S3C). There were 5 m6A regulators that are related to disease-free survival (DFS) in HNSC (Figure 1E and S3D). Patients were ranked according to the expression of m6A regulators with a cut-off value of 50%. High expression of RPUSD3, METTL3, ADAT2, and RPUSD4 was associated with poor prognosis in HNSC. NSUN7, however, had a positive impact on HNSC patient survival. More m6A regulator genes played a role in the survival of CESC (7 genes, including NSUN7, ALKBH8, ZCCHC4, WBSCR22, NSUN4, RPUSD2, and METTL3) patients compared to HNSC. All seven genes had a detrimental effect on the survival of CESC patients (Figure 1E and S3E).

METTL3 was screened by taking the intersection of DFS-related genes in HNSC, DFS-related genes in CESC, 17 overlapping DEGs of CESC and HNSC, and 28 HPV-related DEGs of HNSC (Figure 1G). METTL3 (Methyltransferase 3, N6-Adenosine-Methyltransferase Complex Catalytic Subunit) encodes the 70 kDa subunit of MT-A which is part of N6-adenosine-methyltransferase. This enzyme is involved in the post-transcriptional methylation of internal adenosine residues in eukaryotic mRNAs, forming m6A 34. Patients were ranked according to METTL3 expression, and the top 50% of patients were in METTL3 ^high^ status, otherwise, they were in METTL3^low^ status. METTL3 ^high^ status in HNSC and CESC was related to a poor prognosis.

In addition, the overall survival (OS) maps of m6A regulator genes in multiple cancer types were also plotted (Figure S4A). Only METTL5, RPUSD1, and DKC1 were identified to be associated with OS in HNSC (Figure S4B). METTL5 and DKC1 promoted poor prognosis in HNSC patients. In contrast, high RPUSD1 expression contributed to a better prognosis (Figure S4B). For CESC, three OS-related m6a regulator genes were also identified, namely CTU1, ALKBH5, and METTL14. CTU1 and METTL14 were impairing factors in CESC. In contrast, the high expression of ALKBH5 was a protective factor (Figure S4A and S4C).

### The landscape of genetic variations of m6A regulators in HNSC and CESC

Although both HNSC and CESC were HPV-related squamous cell carcinoma, the mutation spectrums were not the same. Initially, we summarized the distribution of different types of mutations in HNSC and CESC (Figure S5A). Other types of mutations (65.88% samples) and missense substitution (60.14% samples) were the most common mutations in HNSC. Significantly different, missense substitution was observed in 92.74% of CESC samples, which was the most common type of mutation in CESC. The breakdown of the observed substitution mutations was also explored in HNSC and CESC (Figure S5B). In HNSC and CESC, the percentage of each base substitution fluctuated smoothly, with the greatest variation in G>A (65.84% for HNSC and 80.92% for CESC). The types of genomic alterations in HNSC and CESC were analyzed with cbioportal (Figure S5C). The most common type of genomic alteration in HNSC and CESC was amplification. The next in line were mutations in HNSC and deep deletions in CESC, respectively.

Mutation characteristics of m6A regulators in HNSC and CESC were explored with cbioportal. A total of 38 common m6A regulators, including writers, readers, and erasers, were included in the analysis 13. There were similar alteration profiles of these m6A regulators in HNSC and CESC (Figure 1F). The frequency of alterations (altered/profiled) in m6A regulators was not high in either cancer. Exceptionally, deep deletions of RPUSD4, PUS3, ALKBH8, and ADAT3 were more frequent in CESC than in HNSC.

To further explore the characteristics of the samples with m6A regulators alteration, the HNSC and CESC were then divided into altered and unaltered groups based on whether these 38 m6A regulators were altered. Genes with the highest alteration frequency in the altered or unaltered group were listed (Figure S5D). Among the most frequently altered genes in HNSC and CESC, there were only two overlapping genes, namely PIK3CA and TTN. The gap of the alteration event frequency of the listed genes between altered and unaltered groups was much larger in HNSC than in CESC. In both HNSC and CESC, mutation count, fraction genome altered and the MSIsensor score were higher in the altered group compared to the unaltered group, implying higher genomic instability (Figure S5E).

### METTL3 was associated with HPV infection in HPV related cancer

**Figure 2:**
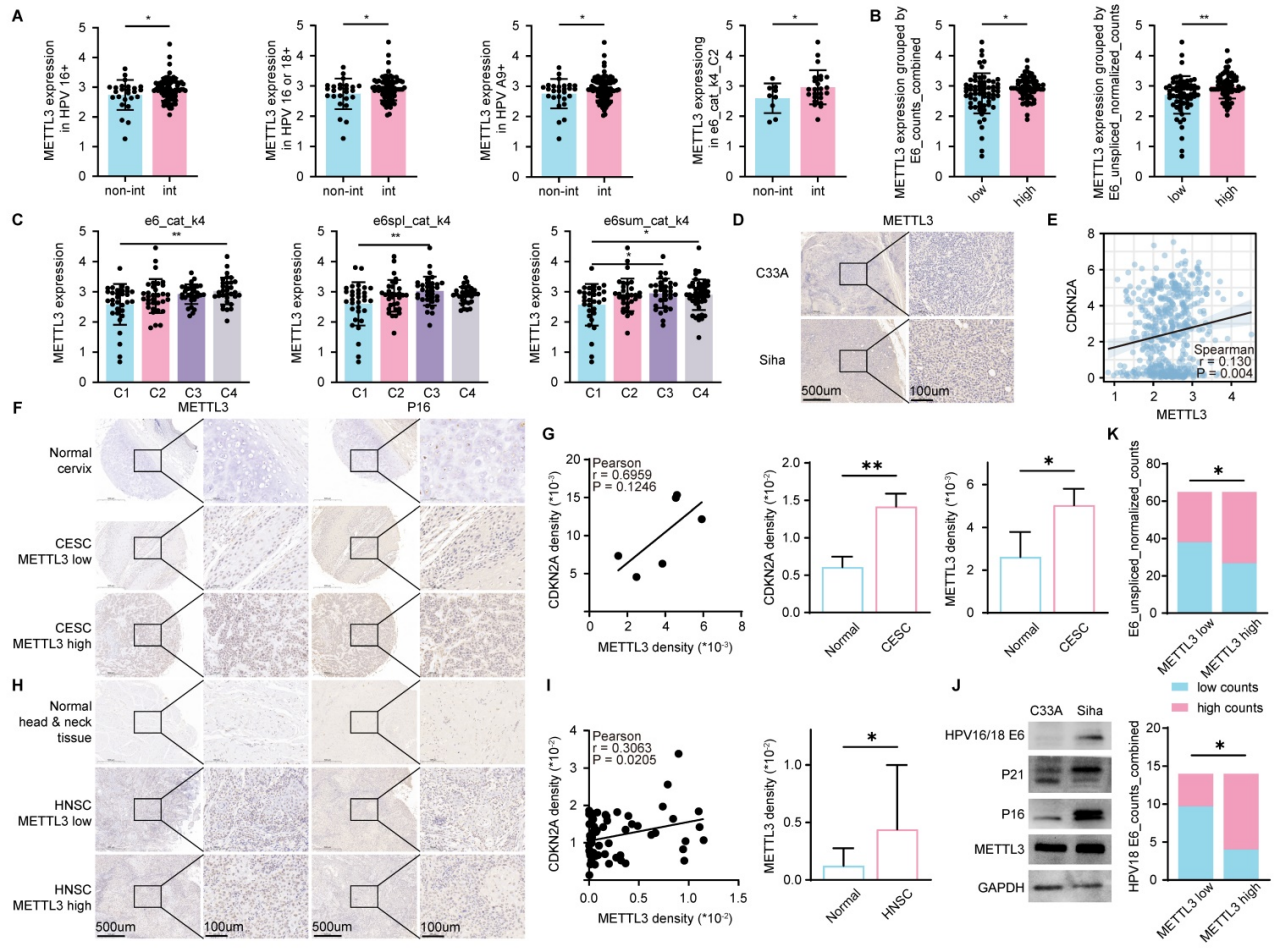
** METTL3 was associated with HPV infection in HPV-related cancer.** (A-C) METTL3 expression in the corresponding group in CESC. (A) Non-Int: without HPV integration, Int: with HPV integration. (B) METTL3 expression in high or low group classified according to CLIN: E6_counts_combined or CLIN: E6_unspliced_normalized_counts. (C) METTL3 expression in different categories of CLIN: e6_cat_k4, CLIN: e6spl_cat_k4, and CLIN: e6sum_cat_k4. (D) IHC staining in C33A (HPV negative CESC cell line) or Siha (HPV positive CESC cell line) derived xenograft subcutaneous tumor. (E) Correlation of CDKN2A expression and METTL3 expression in HNSC. (F-G) Representative images and quantification of IHC staining of CESC samples. (H-I) Representative images and quantification of IHC staining of HNSC samples. (J) Western blot of C33A and Siha. (K) Grouping statistics for HPV E6 and METTL3. P values are indicated as *, P < 0.05; **, P < 0.01; ***, P < 0.001; and ****, P < 0.0001.

We found a high expression of METTL3 in HPV-positive HNSC (Figure 1D). To further illustrate the relationship between HPV infection and METTL3 expression, we used CESC data with detailed HPV infection status 35. In patients with HPV16 positive, HPV16 or HPV18 positive, HPV A9 positive, and CLIN:e6_cat_k4_C2, METTL3 expression was higher in those with HPV integration compared to those without HPV integration (Figure 2A). Patients were classified into high and low groups according to CLIN: E6_counts_combined or CLIN: E6_unspliced_normalized_counts and METTL3 expression was higher in the high group (Figure 2B). The expression of METTL3 varies according to CLIN: e6_cat_k4, CLIN: e6spl_cat_k4, and CLIN: e6sum_cat_k4 (Figure 2C). We then used nude mice to construct cell line-derived xenograft subcutaneous tumor models and found that METTL3 staining was darker in Siha (HPV positive cervical cancer cell line)-derived tumors than in C33A (HPV negative cervical cancer cell line)-derived tumors (Figure 2D).

CDKN2A (p16) was a reported biomarker of HPV 36. Using TCGA HNSC data, METTL3 expression was positively correlated with CDKN2A expression (Figure 2E). To verify the relevance, we used tumor and corresponding normal tissue samples from HNSC and CESC patients, followed by immunohistochemical staining and mean density calculations (Mean density = positive cumulative optical density IOD value/positive area). In the samples, the mean density of CDKN2A was positively correlated with the mean density of METTL3 (P=0.0205 for HNSC and P=0.1246 for CESC, which may be due to the limited sample size of CESC). In CESC, the mean density of CDKN2A in tumor samples was higher than the corresponding normal tissue. Similarly, in CESC and HNSC, the mean density of METTL3 was also higher in tumor samples than in the corresponding normal tissue samples (Figure 2F-I). In addition, the expressions of p21, p16, and METTL3 in the HPV-positive cell line Siha were higher than those in the HPV-negative cell line C33A (Figure 2J). CESC patients were then grouped according to METTL3 expression, and their CLIN: E6_unspliced_normalized_counts and CLIN: E6_counts_combined for HPV18-positive CESC were different (Figure 2K).

### METTL3 was involved in tumor immune response in HPV related cancer

To investigate the role of METTL3 expression levels on HPV-associated tumors, patients were ranked according to METTL3 expression, and the top 50% of patients were in the METTL3 ^high^ group, otherwise, they were in the METTL3 ^low^ group, as previously described. Then, GO/KEGG enrichment analysis was performed on the DEGs between the METTL3 ^high^ group and METTL3 ^low^ group in HNSC and CESC. It has been reported that m6A regulators, including METTL3, regulate immune responses to anti-PD-1 therapy, immune infiltrates, and PD-L1 expression 26, 37, 38. Thus, we focused on the enrichment of DEGs in immune-related pathways. In CESC, enrichment of DEGs can be found in several pathways related to immune responses, including cell recognition, cytokines, immune cell migration and chemotaxis, and humoral immunity (Figure S6A and S6B). Following that, GSEA analysis in CESC was performed to further understand the enrichment of DEGs in immune-related pathways. These DEGs could be found to be significantly associated with signal transduction by growth factor receptors and second messengers, interleukins, IL-10 synthesis, cancer, immunoregulatory interactions between lymphoid and non-lymphoid cells, cytokine-cytokine receptor interaction, and innate immune system (Figure S6C and S6D). Meanwhile, the enrichment of DEGs in immune-related pathways can be found in HNSC, such as receptor-ligand activity and humoral immune response (Figure S6E and S6F). Therefore, METTL3 was involved in tumor immune response in HPV related cancer.

### METTL3 was related to the expression of immune checkpoint molecules in HPV related cancer

**Figure 3:**
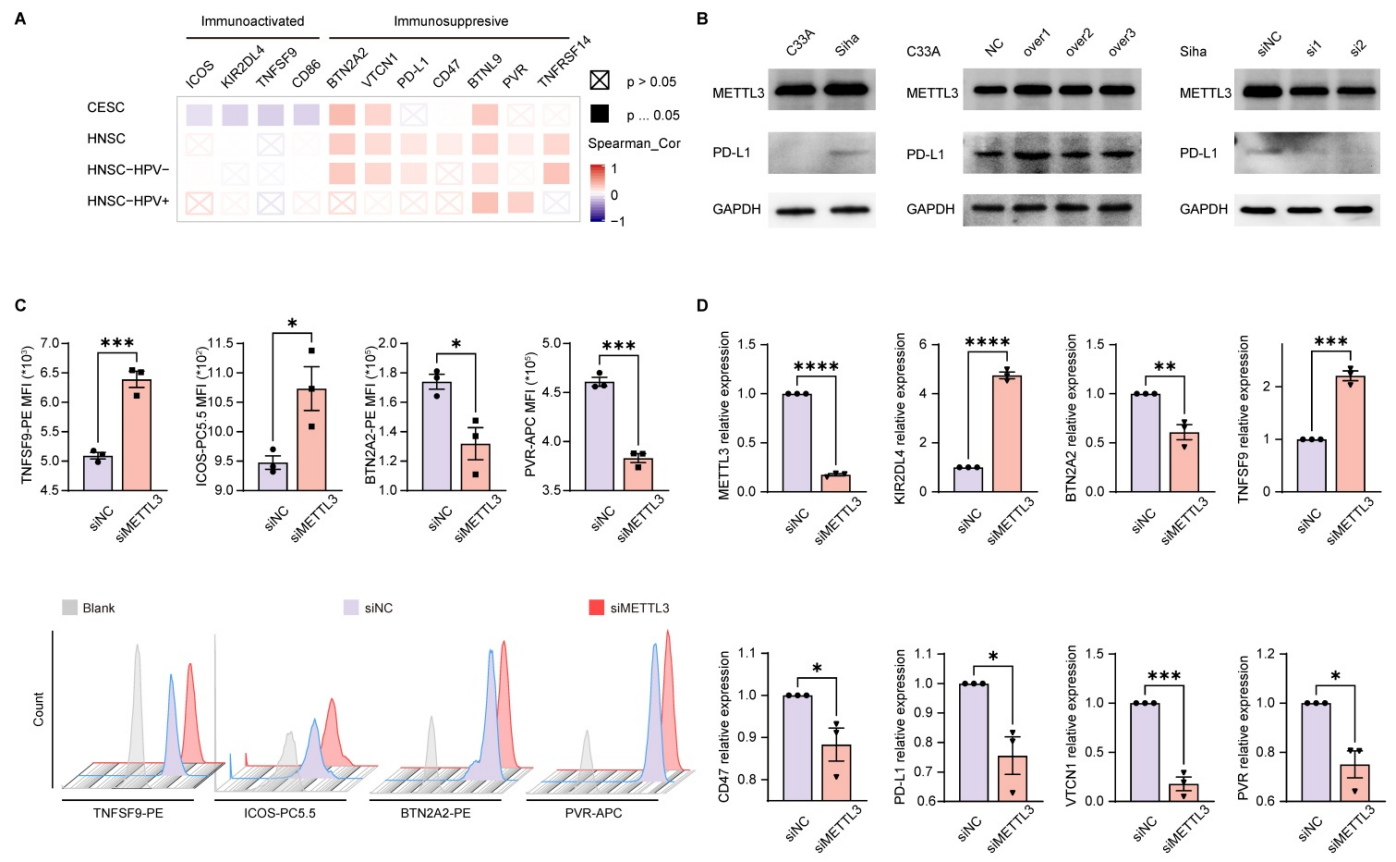
** METTL3 was related to the expression of immune checkpoint molecules in HPV-related cancer.** (A) Heat map of correlation between the expression of immune checkpoint molecules and METTL3 expression in HNSC and CESC was analyzed by TIMER 2.0. (B) Western blot of METTL3 and PD-L1 expression in cell lines. C33A was used to overexpress METTL3 with plasmids, while Siha was used to downregulate METTL3 with siRNA. (C) The immune checkpoint molecules of Siha were detected and quantified by flow cytometry. (D) Relative mRNA expression levels of immune checkpoint molecules in Siha. P values are indicated as *, P < 0.05; **, P < 0.01; ***, P < 0.001; and ****, P < 0.0001.

**Figure 4:**
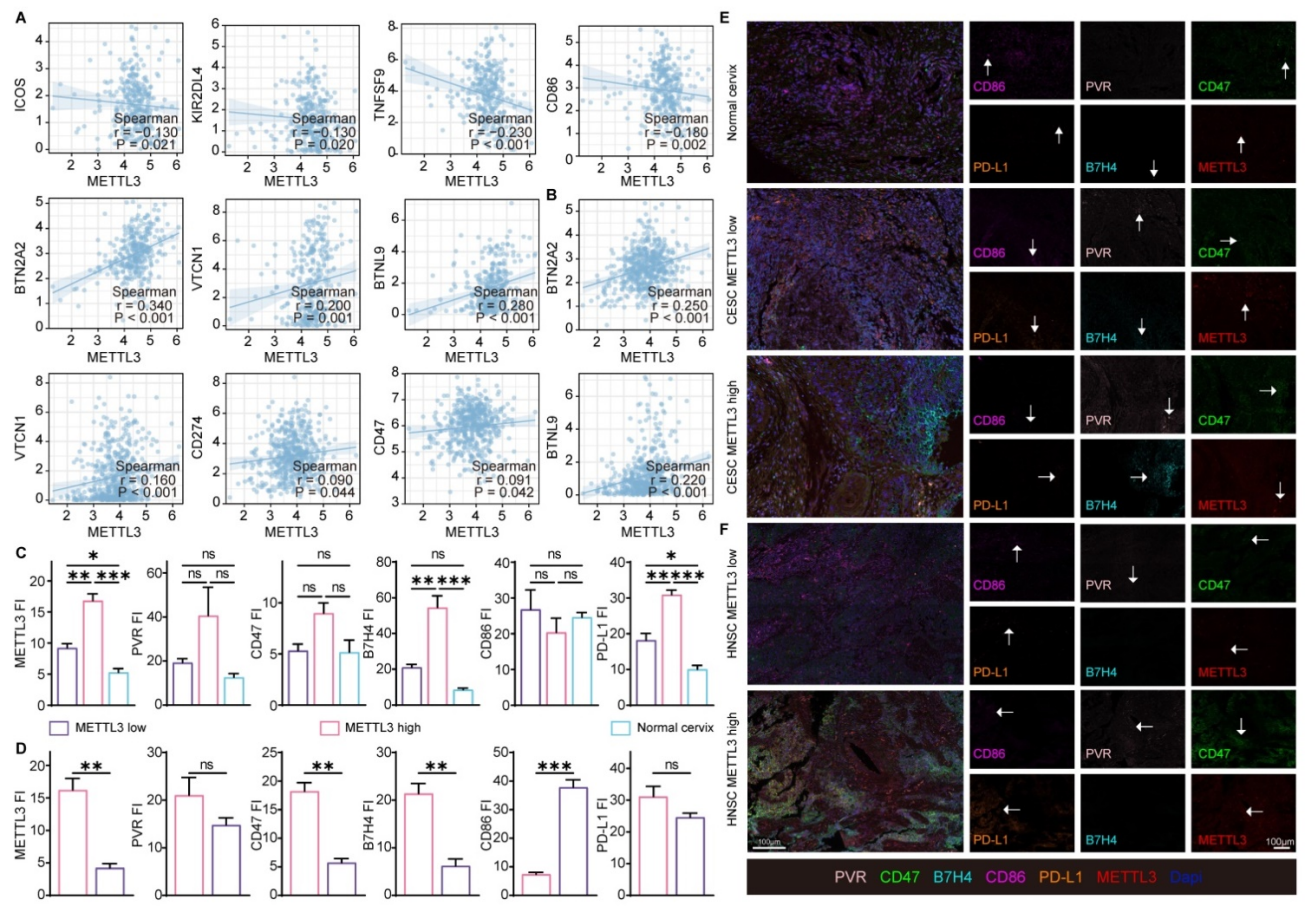
** METTL3 correlated with the expression of immune checkpoint molecules in samples.** (A) Correlation of immune checkpoint molecule expression with METTL3 expression in CESC. (B) Correlation of immune checkpoint molecule expression with METTL3 expression in HNSC. (C-E) Representative images of multispectral fluorescence immunohistochemistry of paired samples and their quantification. P values are indicated as *, P < 0.05; **, P < 0.01; ***, P < 0.001; and ****, P < 0.0001.

The above enrichment analysis suggested that METTL3 was associated with cell migration, chemotaxis, and cytokines, which played a role in the immune response. To investigate how METTL3 affected the immune response, we analyzed the relationship between immune checkpoint molecules 39 and METTL3 expression. ICOS, KIR2DL4, TNFSF9, and CD86 played a role in immune activation and negatively correlated with METTL3 expression in CESC. Conversely, BTN2A2, VTCN1, PD-L1, CD47, BTNL9, PVR, and TNFRSF14 were immune-activating molecules that showed a positive correlation with METTL3 expression levels in HNSC. It was worth noting that BTN2A2, VTCN1, and BTNL9 had such trends as above in both HNSC and CESC (Figure 3A and Figure 4A).

To verify the above results, we used C33A, an HPV-negative cervical cancer cell line, and Siha, an HPV-positive cervical cancer cell line. METTL3 expression in the HPV-positive cell line (Siha) is higher than the HPV-negative cell line (C33A). Meanwhile, the expression of PD-L1 in Siha was higher than that in C33A. Following that, C33A was used to overexpress METTL3 with plasmid and Siha was used to knock down METTL3 with siRNA. After METTL3 overexpression, PD-L1 expression was also elevated in C33A. In Siha, down-regulation of METTL3 was accompanied by high expression of PD-L1 (Figure 3B). We also verified these correlations using flow cytometry. METTL3 down-regulation promoted TNFSF9 and ICOS expression and inhibited BTN2A2 and PVR expression in Siha (Figure 3C). This correlation was also verified at the transcriptional level. Reduced expression of METTL3 was accompanied by upregulation of KIR2DL4 and TNFSF9 and downregulation of BTN2A2, CD47, PD-L1, VTCN1, and PVR (Figure 3D). Besides, overexpression of METTL3 in Siha inhibited CD86 expression and promoted BTN2A2 expression (Figure S7A).

Using TCGA data, correlation analyses of the expression of these immune checkpoint molecules and METTL3 in HNSC and CESC were performed (Figure 4A-4B, and Figure S7B). Multispectral immunohistochemical staining of paired samples of patient origin was performed to observe these relationships. Patients were ranked according to the fluorescence index (FI) of METTL3, and the top 50% of patients were METTL3 ^high^ status, otherwise, they were METTL3 ^low^ status. In CESC samples, METTL3 was overexpressed in cancer compared with the normal cervix (Figure 4C and 4E). In HNSC and CESC, METTL3 ^high^ samples showed lower PVR, CD47, B7H4, and PD-L1 FI and higher CD86 FI compared with METTL3 ^low^ samples (Figure 4C-4F).

### METTL3 impacts on immune cell infiltration in HPV related cancer

**Figure 5:**
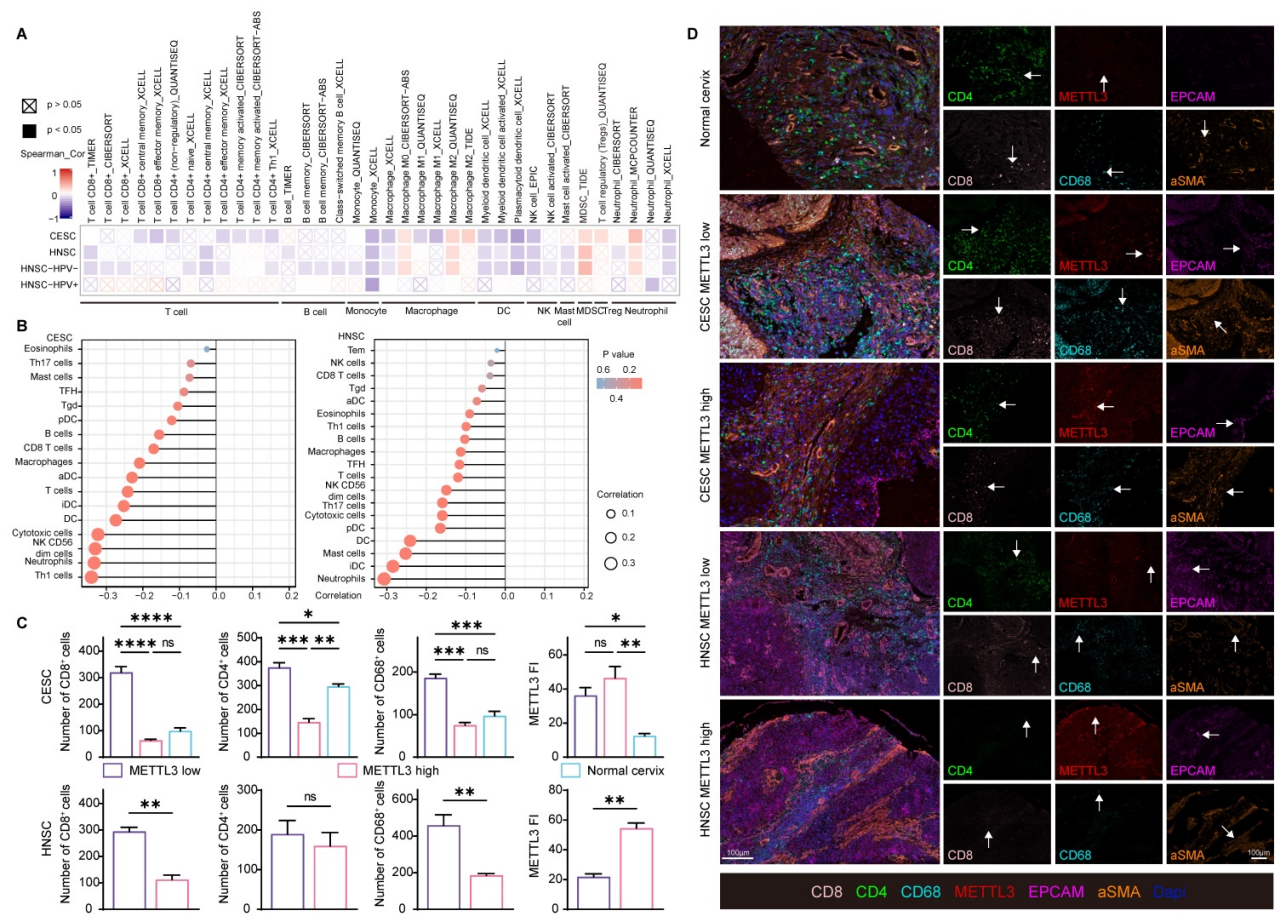
** METTL3 impacts on immune cell infiltration in HPV-related cancer.** (A) Heat map of correlation between the expression of immune cell infiltration levels and METTL3 expression in HNSC and CESC was analyzed by TIMER 2.0. (B) Lollipop plot of immune cell infiltration analysis of HNSC and CESC using ssGSEA of GSVA R package (version 1.34.0) based on TCGA data. (C-D) Representative images of multispectral fluorescence immunohistochemistry of paired samples and their quantification. P values are indicated as *, P < 0.05; **, P < 0.01; ***, P < 0.001; and ****, P < 0.0001.

Besides immune checkpoint molecules, to investigate how METTL3 affected the immune response, immune cell infiltration was also analyzed in HPV-related cancer. METTL3 was negatively related to CD8^+^ and CD4^+^ T cells, B cells, monocytes, macrophage M1, dendritic cells, natural killer cells, and mast cells infiltration. The infiltration of macrophage M2, myeloid-derived suppressor cells, and regulatory T cells showed a positive correlation with METTL3 expression (Figure 5A and Figure S8). An alternative algorithm was used to verify the above results (Figure 5B and Figure S9).

To verify these correlations, we used paired samples from HNSC and CESC patients for multispectral immunohistochemical staining and quantitation. Patients were ranked according to the FI of METTL3, and the top 50% of patients were METTL3 ^high^ status, otherwise, they were METTL3 ^low^ status, as previously described. In CESC, METTL3 FI was higher in tumor tissue than in the normal cervix. In HNSC and CESC, METTL3 ^high^ status was associated with low infiltration of CD8^+^, CD4^+^ and CD68^+^ cells (Figure 5C and 5D).

In addition to CESC and HNSC, we also explored the role of METTL3 in oral squamous cell carcinoma (OSCC), a tumor in which some patients are infected with HPV 40, 41. The expression of METTL3 was not associated with prognosis of OSCC, including progression-free survival and overall survival (Figure S10A and S10B). But the expression of METTL3 showed diagnostic value for OSCC (Figure S10C). In the tumor immune microenvironment, METTL3 was negatively correlated with immune cells such as NK cells, B cells, T cells, DCs, and macrophages. However, it also showed a negative correlation between METTL3 and Treg. Furthermore, METTL3 was associated with increased T helper cell and Tcm infiltration (Figure S10D). Therefore, the effect of METTL3 on immune cell infiltration in OSCC is inconsistent. The expression of METTL3 was positively correlated with the expression of immunosuppressive molecules VTCN1, BTN2A2, CD47, BTNL9 and PVR (Figure S10E and S10F). However, the role of HPV in OSCC and the correlation between the two was not clear 40, 41, which might be one of the reasons why METTL3 was not associated with OSCC prognosis.

### METTL3 inhibitor in combination with anti-PD-1 therapy inhibited tumor progression *in vivo*

**Figure 6:**
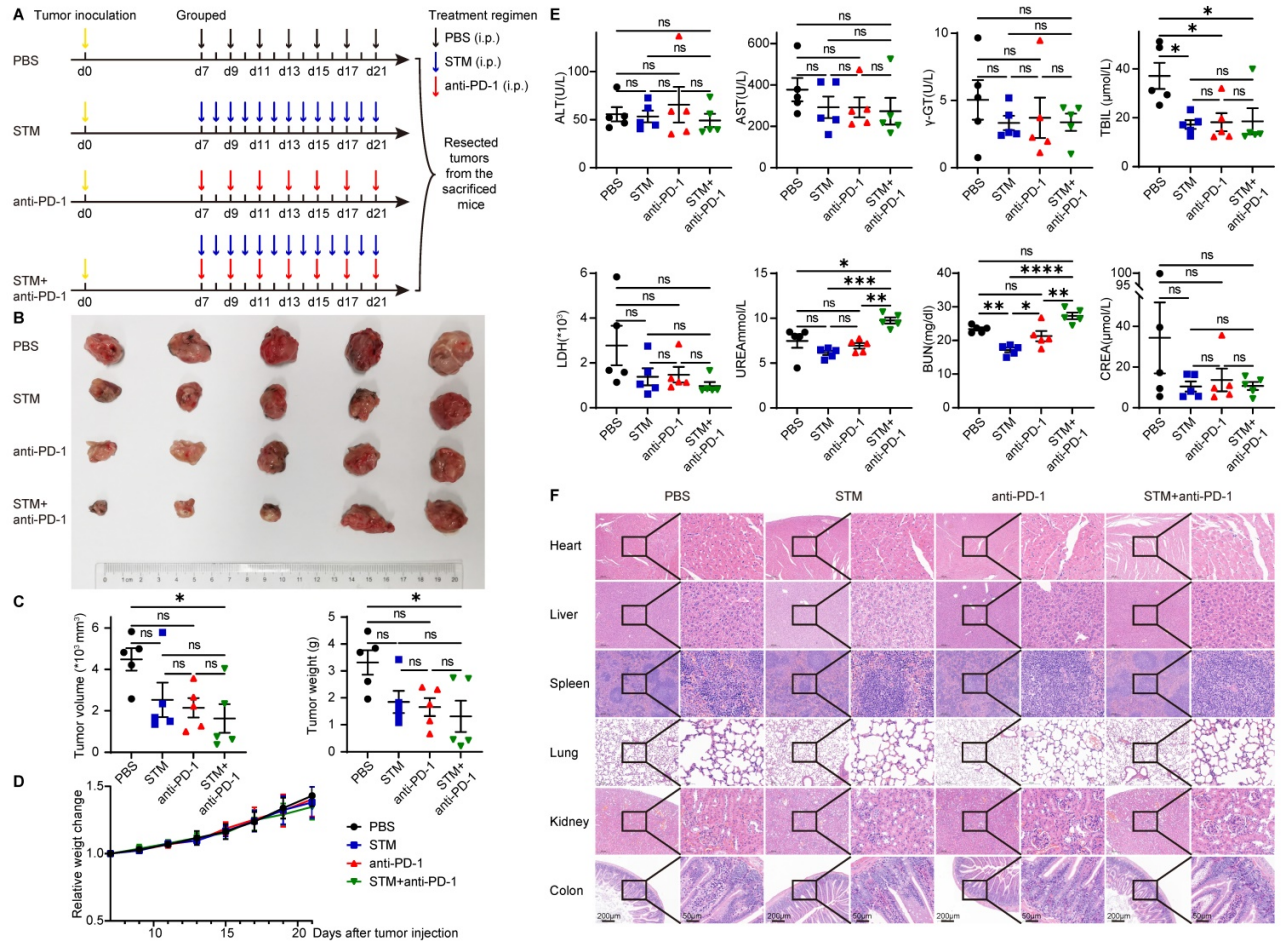
** METTL3 inhibitor in combination with anti-PD-1 therapy inhibits tumor progression *in vivo.*** (A) Animal experiment model diagram. Seven days after U14 injection, mice were randomly assigned to four groups and given PBS (every two days), STM2457 (once daily), anti-PD-1 (once daily), and STM2457+anti-PD-1 treatments. After two weeks of continuous administration, the mice were killed and their tumors were excised. (B) Photo of the excised tumor. (C) Volume and weight of the excised tumors. (D) Bodyweight change curve of mice. (E) Levels of liver and kidney function indicators in plasma. (F) HE staining of organs. P values are indicated as *, P < 0.05; **, P < 0.01; ***, P < 0.001; and ****, P < 0.0001.

STM2457 is a highly potent and selective METTL3 inhibitor, which could be a potential therapeutic strategy against acute myeloid leukemia 42. To investigate the role of METTL3 inhibitor and immune therapy in cancer, U14 was injected subcutaneously into C57BL/6 mice. Seven days after injection, mice were randomly assigned to four groups and given PBS (every two days), STM2457 (once daily), anti-PD-1 (once daily), and STM2457+anti-PD-1 treatments (Figure 6A). After two weeks of continuous administration, the mice were killed and their tumors were excised. Tumor volume and tumor weight in the STM2457+anti-PD-1 treatment group were significantly smaller than those in the other three groups. There was no significant effect of STM2457 treatment alone or anti-PD-1 treatment alone compared to the PBS group (Figure 6B and 6C).

As for the safety of these therapies, there was no significant difference in the bodyweight of the mice in the remaining three groups compared to the PBS group (Figure 6D). Meanwhile, plasma biochemical indicators reflecting liver and kidney functions, such as ALT, AST, γ-GT, TBIL, LDH, UREA, BUN, and CREA, were not significantly elevated compared with the PBS group (Figure 6E). Organs such as the heart, liver, spleen, lung, kidney, and intestine showed no significant histological changes (Figure 6F).

## Discussion

Current therapeutic approaches of HPV-associated cancers urgently require the discovery of precise and effective targets underlying HPV-host interactions 8, 9, 43. The key finding of this study was that m6A regulator METTL3 was associated with tumor HPV status and HPV expression, and METTL3^ high^ status facilitated the formation of an immunosuppressed tumor microenvironment and targeting METTL3 promoted immunotherapy in HPV-related cervical cancer.

m6A RNA modifications participate in tumor proliferation, differentiation, tumorigenesis, proliferation, invasion, and metastasis, and function as oncogenes or anti-oncogenes in malignant tumors 15. Here, we identified METTL3, an m6A RNA methyltransferase, over-expressed in both HNSC and CESC, associated with poor prognosis. Zhao et al. indicated that m6A regulators including METTL3 have the role of predicting prognosis in HNSC patients 44. Also, METTL3 was found to be consistently upregulated, and high METTL3 expression was associated with the prognosis of oral squamous cell carcinoma patients. It is also reported that METTL3 overexpression promotes the proliferation and migration of hepatocellular carcinoma cells 45. Besides, m6A modification in cancer treatment indicates new directions for the treatment of various cancers 15. Pancreatic cancer with downregulated METTL3 expression is reported to be sensitive to anticancer drugs and radiotherapy 44. STM2457, a highly potent and selective METTL3 inhibitor, was reported to be a potential therapeutic strategy against acute myeloid leukemia 42. As for cervical cancer, STM2457 in combination with anti-PD-1 therapy inhibited tumor progression *in vivo.* Regulators or inhibitors of m6A modifications may provide potential therapeutic strategies for cancers 15.

So far, few studies have described the relationship between HPV and m6A methylation 20. Interestingly, in our research, METTL3 overexpression was found to be related to HPV status, linking m6A modification and HPV infection. Zhao et. al. demonstrated that circE7 possesses m6A modifications in the cytoplasm, and is translated to produce E7, an oncoprotein, yielding novel insights into how HPV regulates infection and tumorigenesis 19. Besides, in terms of HPV-related cancer, four regulators (RBM15, METTL3, FTO, and YTHDF2) were identified to be aberrantly expressed in CESC tissues, and ZC3H13, YTHDC1, and YTHDF1 formed a prognostic indicator 46. Similarly, m6A modifications play a role in the progression and immune response of HNSC, another HPV-associated cancer 26.

Increasing evidence demonstrated that m6A modification took on an indispensable role in antitumor immunity through interactions with various m6A regulators 21, 47, 48. m6A modification was reported to promote PD-L1 expression in HNSC 26. In our research, overexpression of METTL3 was associated with upregulation of immunosuppressive immune checkpoint molecules, and downregulation of immune activated immune checkpoint molecules. A low m6A score was linked to increased neoantigen load and enhanced response to anti-PD-1/L1 immunotherapy in gastric cancer 21. Consistent with this, inhibition of m6A modification enhances the response of colorectal cancer and melanoma to immunotherapy 37. Nevertheless, it was reported that METTL3-mediated m6A RNA methylation promotes the anti-tumor immunity of natural killer cells 49. Besides, the therapeutic efficacy of PD-1 checkpoint blockade is attenuated in Mettl3-deficient mice 48. Therefore, the role of m6A modifications in immunotherapy needs further investigation.

We also revealed that METTL3 inhibited the infiltration of immune cells, playing an immunomodulatory role in HPV-associated cancers. The m6A regulator-based risk signatures and m6A modification patterns were identified to be associated with the immune cell infiltration levels of patients with HNSC, gastric cancer, hepatocarcinoma, and adrenocortical carcinoma 21, 26, 50, 51. Mellt3- deficiency could increase cytotoxic tumor‐infiltrating CD8^+^ T cells and elevate secretion of IFNγ, Cxcl9, and Cxcl10 in colorectal tumor 52. In addition, our study found that METTL3 was related to HPV infection and the expression of immunosuppressive immune checkpoint molecules, which provides insights for the immunotherapy of HPV-related tumors. Ablation of Mettl3 in myeloid cells increases M1/M2-like tumor-associated macrophage and regulatory T cell infiltration into tumors, promoting tumor growth and metastasis *in vivo* 48. These researches confirmed the immunomodulatory role of METTL3. However, there is still some unresolved knowledge regarding the relationship between m6A modifications and anti-cancer immune responses, and identifying the role of distinct m6A modification patterns in the tumor microenvironment at a more refined level with new techniques will enhance our understanding of antitumor immune response, and provide promising immunotherapy strategies 21, 26, 48. Although studies found the immunoregulatory role of METTL3 in tumors, we not only identified a role for METTL3 in immunity, but also found a relationship between METTL3 and HPV infection or HPV-related tumors.

Despite the positive aspects, there were some limitations in our research. How HPV infection affects the expression of METTL3, and the mechanism by which METTL3 affects the expression of HPV infection-related molecules such as p16 needs to be further elucidated. In addition, we have not studied the molecular mechanism of METTL3-mediated immunosuppression.

## Conclusions

In this study, by integrative trans-omic analyses of m6A regulators in HNSC and CESC, we identified the key m6A regulator METTL3 in tumor HPV status, HPV expression, suppressive TIME, and suppressive immune checkpoint molecules in HPV-associated cancers. Furthermore, METTL3 inhibitor combined with anti-PD1 therapy promoted immunotherapy of CESC *in vivo*, which could be a potential therapeutic target in anticancer immunotherapy.

## Supplementary Material

Supplementary figures and table.

## Abbreviations

HNSC: head and neck squamous cell carcinoma
CESC: cervical squamous cell carcinoma
HPV: human papillomavirus
m6A: N6-methyladenosine
TIME: tumor immune microenvironment
GO: Gene ontology
KEGG: Kyoto Encyclopedia of Genes and Genomes
ALT: Alanine transaminase
AST: aspartate aminotransferase
γ-GT: gamma-glutamyl transferase
TBIL: total bilirubin
LDH: lactate dehydrogenase
BUN: blood urea nitrogen
CREA: creatinine concentration
DFS: disease-free survival
OS: overall survival
DEGs: differentially expressed genes
FI: fluorescence index

## Highlights

m6A regulator expression and variation spectrum in HPV-related cancers.

METTL3 was an HPV status-associated m6A regulator.

METTL3 ^high^ status was negatively correlated with tumor immune cell infiltrations.

METTL3 ^high^ status facilitated immunosuppressive immune checkpoint molecules expression.

STM2457 in combination with anti-PD-1 therapy inhibited cervical cancer progression *in vivo.*
