# Supplementary material for: Integrative Analyses of m6A Regulators Identify that METTL3 is Associated with HPV Status and Immunosuppressive Microenvironment in HPV-related Cancers
Source: Int J Biol Sci. 2022 Jun 3;18(9):3874–87. doi: 10.7150/ijbs.70674 (PMC9254478; doi:10.7150/ijbs.70674)
Supplement: Supplementary file 1 — Supplementary figures and table. [file ijbsv18p3874s1.pdf]

Figure S1. The expression of m6A related genes in CESC and HNSC and their location.

(A) Venn of differentially expressed m6A regulators in tumor tissue and normal tissue in CESC and HNSC. (B) Expression levels of the 17 shared differentially expressed m6A regulators. (C-D) The location of DEGs in HNSC and CESC on chromosomes.

Figure S2. Expression levels of m6A regulators in HPV-positive and negative HNSC.

Figure S3. Expression spectrum of m6A regulators in HPV-positive and negative CESC and survival analysis in HPV-related cancer.

(A) Expression profiles of m6A regulators in HPV-positive and negative CESC. (B) Venn diagram of m6A regulators differentially expressed in tumor tissue and normal tissue in CESC and HNSC and HPV-positive and negative HNSC. (C) DFS survival map of m6A regulators. (D) Kaplan-Meier curve of m6A regulators in HNSC. (E) Kaplan-Meier curve of m6A regulators in CESC.

Figure S4. Overall survival analysis of m6A regulators in HPV-related cancer.

(A)Overall survival map of m6A regulators. (B) Kaplan-Meier curve of m6A regulators in HNSC. (C) Kaplan-Meier curve of m6A regulators in CESC.

Figure S5. The landscape of genetic variations of m6A regulators in HNSC and CESC.

(A) Overview of the types of mutation observed. (B) Breakdown of the observed substitution mutations. (C) Alteration frequency in HNSC and CESC. (D) Patients were divided into two groups with or without alterations on the m6A regulators. The

graph shows genes with the highest frequency in any group. (E) Mutation count, fraction genome altered, and MSIsensor scores in any group.

Figure S6. METTL3 was involved in tumor immune response in HPV-related cancer.

Patients were ranked according to METTL3 expression, and the top 50% of patients were in METTL3 ^{high} status, otherwise, they were in METTL3^{low} status. Enrichment analysis and visualization of the filtered DEGs were performed with clusterProfiler, org.Hs.eg.db, and the ggplot2 R package (R 3.6.3). (A-B) Enrichment analysis of differential genes in CESC. (C-D) GSEA analysis of differential genes in immune response-related pathways. (E-F) Enrichment analysis of differential genes in HNSC.

Figure S7. Correlation of immune checkpoint molecules with METTL3 expression in HPV-related cancer.

(A) Relative mRNA expression level in C33A. (B) Correlation of immune checkpoint molecules and METTL3 expression in HNSC. P values are indicated as *, P < 0.05; **, P < 0.01; ***, P < 0.001; and ****, P < 0.0001.

Figure S8. Correlation of immune cell infiltration with METTL3 expression in HPV-associated cancers.

(A) Correlation analysis of the number of immune cells and METTL3 expression in CESC using TIMER 2.0. (B) Correlation analysis of the number of immune cells and METTL3 expression in HNSC using TIMER 2.0.

Figure S9. Immune cell infiltration levels in HPV-related cancer.

Patients were ranked according to METTL3 expression, and the top 50% of patients

were in METTL3 ^{high} status, otherwise, they were in METTL3^{low} status. (A) The level of immune cell infiltration in CESC. (B) The level of immune cell infiltration in HNSC. P values are indicated as *, P < 0.05; **, P < 0.01; ***, P < 0.001; and ****, P < 0.0001.

Figure S10. Clinical and immune relevance of METTL3 to OSCC.

(A-B) The relationship between METTL3 and the prognosis of OSCC. (C) The diagnostic predictive value of METTL3 in OSCC. (D) Correlation between METTL3 expression and immune cells infiltration. (E-F) Correlation between the expression of immune checkpoint molecules and the expression of METTL3.

Supplementary Table 1: Sequence of primers used in real-time PCR.

Figure S1

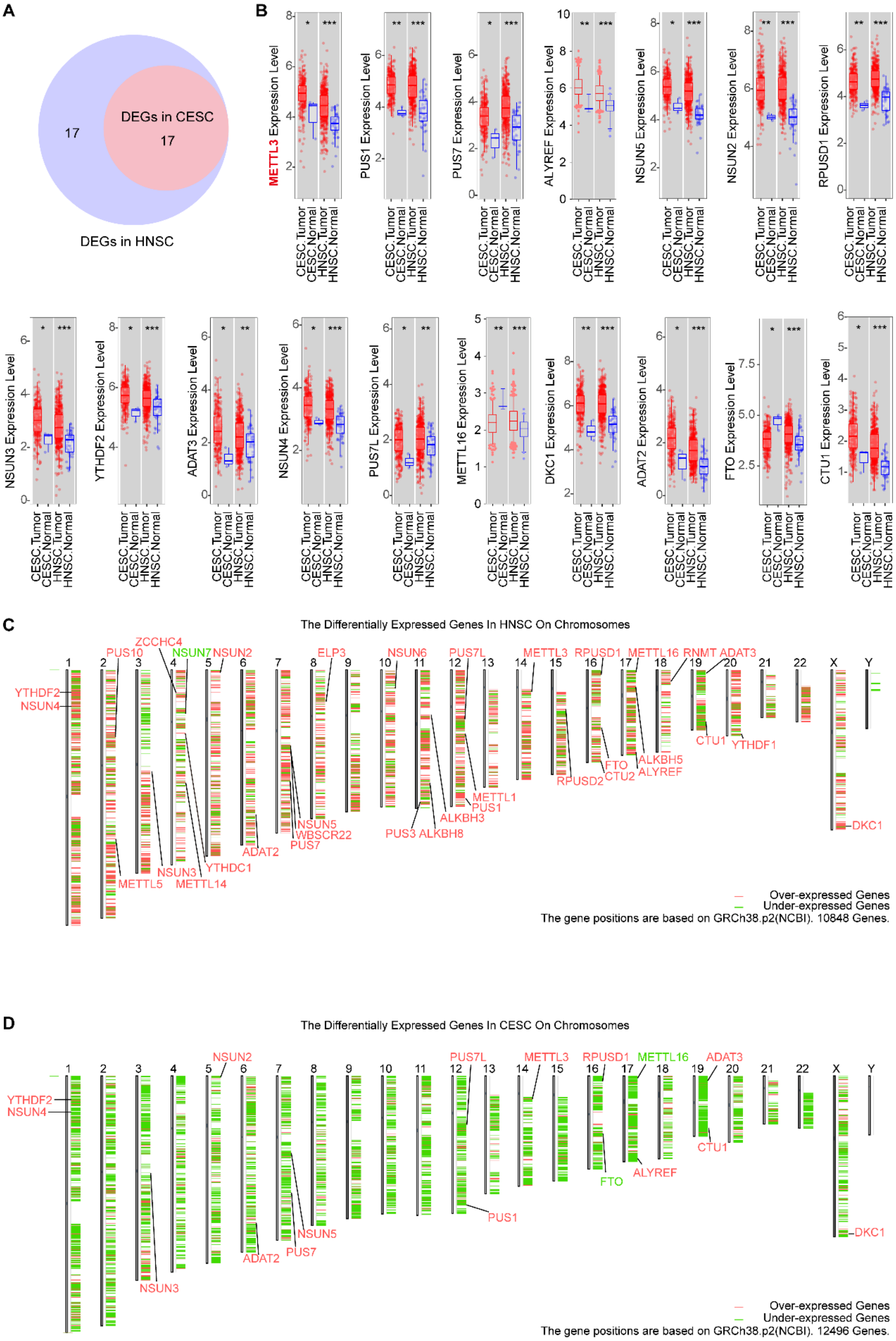
A
DEGs in CESC
17
17
DEGs in HNSC
B
METTL3 Expression Level
PUS1 Expression Level
PUS7 Expression Level
ALYREF Expression Level
NSUN5 Expression Level
NSUN2 Expression Level
RPUSD1 Expression Level
NSUN3 Expression Level
YTHDF2 Expression Level
ADAT3 Expression Level
NSUN4 Expression Level
PUS7L Expression Level
METTL16 Expression Level
DKC1 Expression Level
ADAT2 Expression Level
FTO Expression Level
CTU1 Expression Level
CESC.Tumor
CESC.Normal
HNSC.Tumor
HNSC.Normal
C
The Differentially Expressed Genes In HNSC On Chromosomes
YTHDF2
NSUN4
PUS10
ZCCHC4
NSUN7
NSUN2
ELP3
NSUN6
PUS7L
METTL3
RPUSD1
METTL16
RNMT
ADAT3
CTU1
YTHDF1
FTO
CTU2
ALKBH5
ALYREF
RPUSD2
METTL1
PUS1
ALKBH3
PUS3
ALKBH8
NSUN5
WBSCR22
PUS7
ADAT2
NSUN3
YTHDC1
METTL5
METTL14
DKC1
Over-expressed Genes
Under-expressed Genes
The gene positions are based on GRCh38.p2(NCBI). 10848 Genes.
D
The Differentially Expressed Genes In CESC On Chromosomes
YTHDF2
NSUN4
NSUN2
PUS7L
METTL3
RPUSD1
METTL16
ADAT3
CTU1
FTO
ALYREF
PUS1
NSUN5
PUS7
ADAT2
NSUN3
DKC1
Over-expressed Genes
Under-expressed Genes
The gene positions are based on GRCh38.p2(NCBI). 12496 Genes.

Figure S2

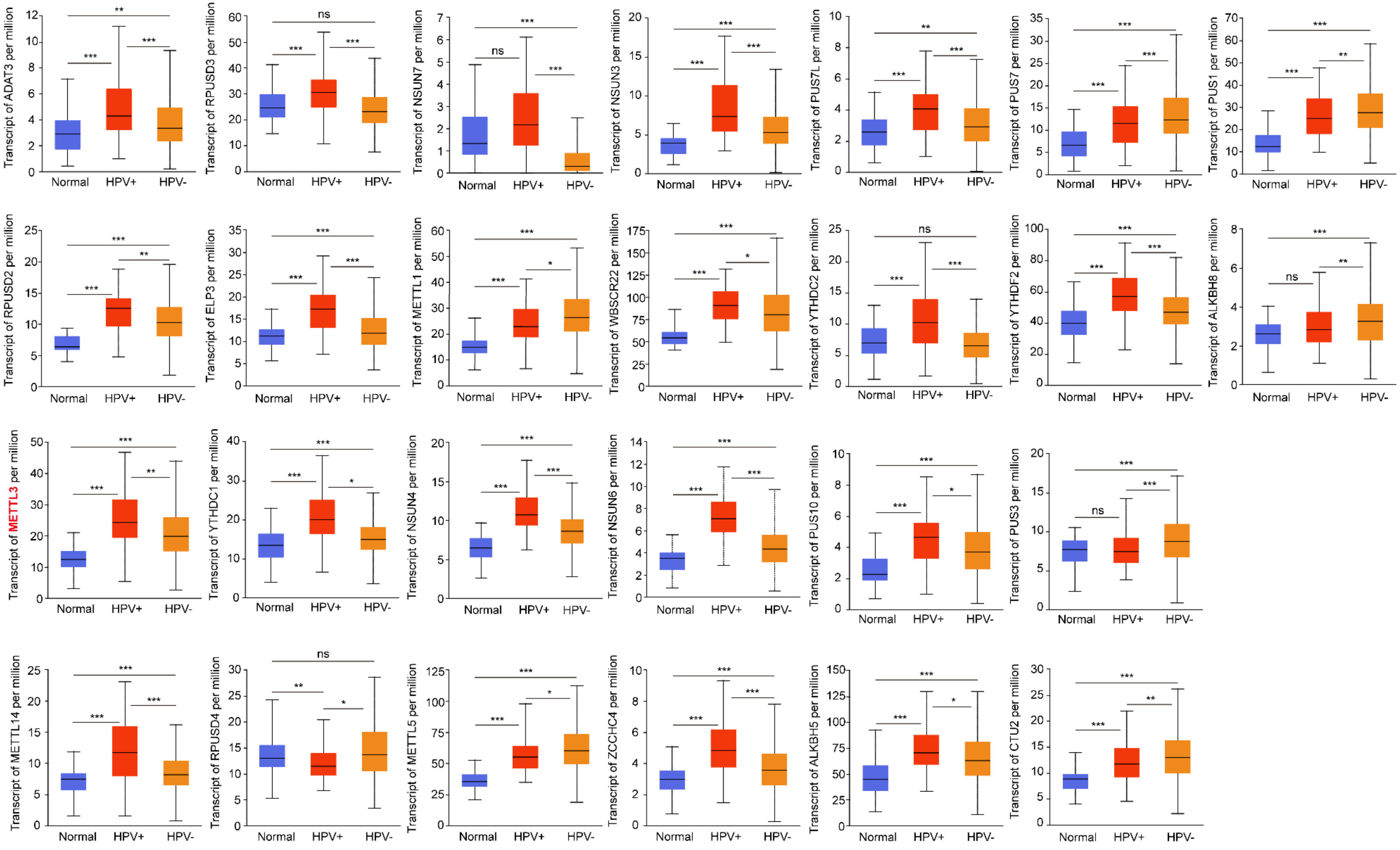

Transcript of ADAT3 per million
Transcript of RPUSD3 per million
Transcript of NSUN7 per million
Transcript of NSUN3 per million
Transcript of PUS7L per million
Transcript of PUS7 per million
Transcript of PUS1 per million
Transcript of RPUSD2 per million
Transcript of ELP3 per million
Transcript of METTL1 per million
Transcript of WBSCR22 per million
Transcript of YTHDC2 per million
Transcript of YTHDF2 per million
Transcript of ALKBH8 per million
Transcript of METTL3 per million
Transcript of YTHDC1 per million
Transcript of NSUN4 per million
Transcript of NSUN6 per million
Transcript of PUS10 per million
Transcript of PUS3 per million
Transcript of METTL14 per million
Transcript of RPUSD4 per million
Transcript of METTL5 per million
Transcript of ZCCHC4 per million
Transcript of ALKBH5 per million
Transcript of CTU2 per million
Normal
HPV+
HPV-

Figure S3

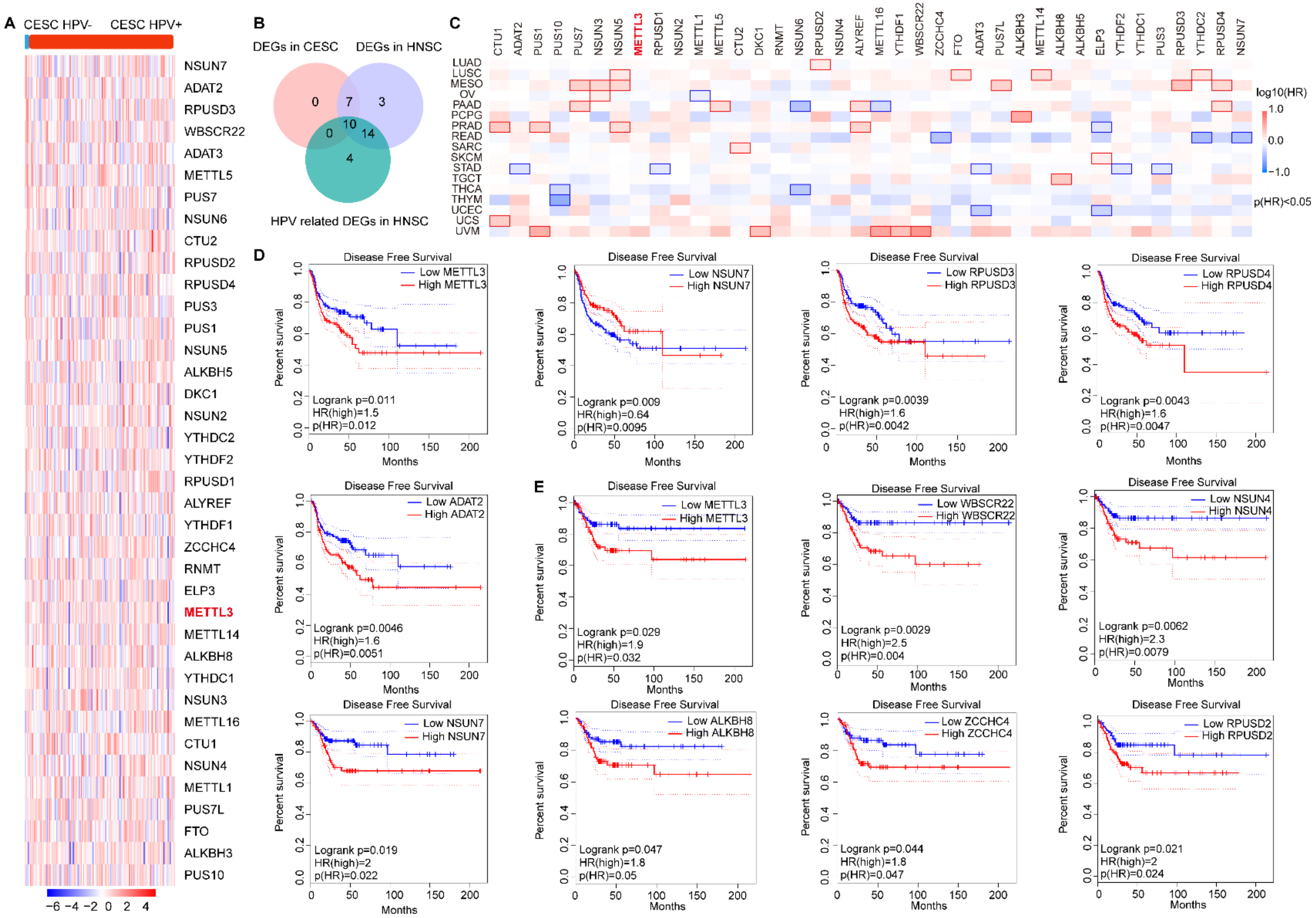
A
CESC HPV-
CESC HPV+
NSUN7
ADAT2
RPUSD3
WBSCR22
ADAT3
METTL5
PUS7
NSUN6
CTU2
RPUSD2
RPUSD4
PUS3
PUS1
NSUN5
ALKBH5
DKC1
NSUN2
YTHDC2
YTHDF2
RPUSD1
ALYREF
YTHDF1
ZCCHC4
RNMT
ELP3
METTL3
METTL14
ALKBH8
YTHDC1
NSUN3
METTL16
CTU1
NSUN4
METTL1
PUS7L
FTO
ALKBH3
PUS10
−6 −4 −2 0 2 4
B
DEGs in CESC
DEGs in HNSC
HPV related DEGs in HNSC
C
LUAD
LUSC
MESO
OV
PAAD
PCPG
PRAD
READ
SARC
SKCM
STAD
TGCT
THCA
THYM
UCEC
UCS
UVM
log10(HR)
p(HR)<0.05
D
E
Disease Free Survival
Percent survival
Months
Low METTL3
High METTL3
Logrank p=0.011
HR(high)=1.5
p(HR)=0.012
Low NSUN7
High NSUN7
Logrank p=0.009
HR(high)=0.64
p(HR)=0.0095
Low RPUSD3
High RPUSD3
Logrank p=0.0039
HR(high)=1.6
p(HR)=0.0042
Low RPUSD4
High RPUSD4
Logrank p=0.0043
HR(high)=1.6
p(HR)=0.0047
Low ADAT2
High ADAT2
Logrank p=0.0046
HR(high)=1.6
p(HR)=0.0051
Low METTL3
High METTL3
Logrank p=0.029
HR(high)=1.9
p(HR)=0.032
Low WBSCR22
High WBSCR22
Logrank p=0.0029
HR(high)=2.5
p(HR)=0.004
Low NSUN4
High NSUN4
Logrank p=0.0062
HR(high)=2.3
p(HR)=0.0079
Low NSUN7
High NSUN7
Logrank p=0.019
HR(high)=2
p(HR)=0.022
Low ALKBH8
High ALKBH8
Logrank p=0.047
HR(high)=1.8
p(HR)=0.05
Low ZCCHC4
High ZCCHC4
Logrank p=0.044
HR(high)=1.8
p(HR)=0.047
Low RPUSD2
High RPUSD2
Logrank p=0.021
HR(high)=2
p(HR)=0.024

Figure S4

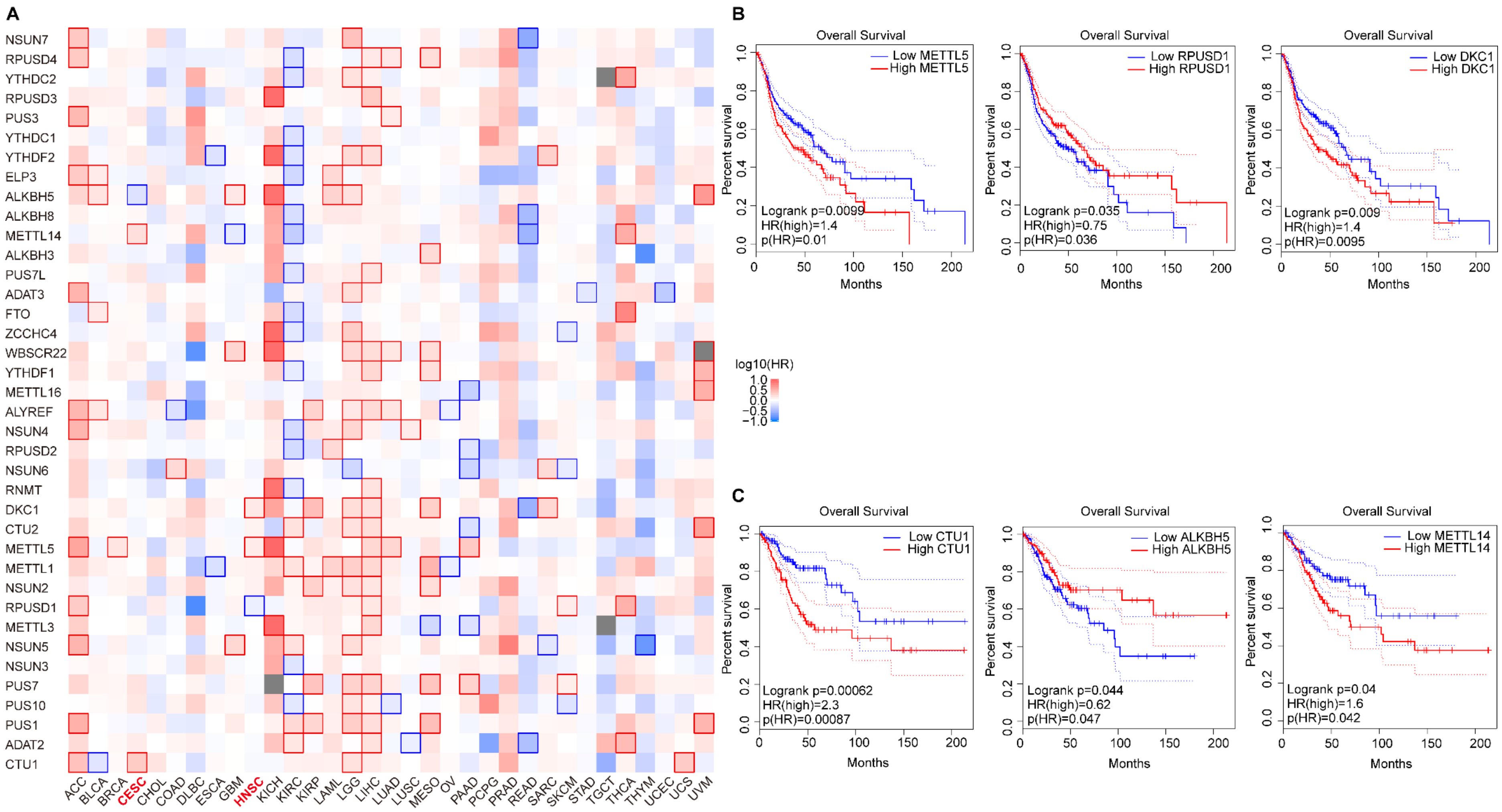
A
NSUN7
RPUSD4
YTHDC2
RPUSD3
PUS3
YTHDC1
YTHDF2
ELP3
ALKBH5
ALKBH8
METTL14
ALKBH3
PUS7L
ADAT3
FTO
ZCCHC4
WBSCR22
YTHDF1
METTL16
ALYREF
NSUN4
RPUSD2
NSUN6
RNMT
DKC1
CTU2
METTL5
METTL1
NSUN2
RPUSD1
METTL3
NSUN5
NSUN3
PUS7
PUS10
PUS1
ADAT2
CTU1
ACC BLCA BRCA CESC CHOL COAD DLBC ESCA GBM HNSC KICH KIRC KIRP LAML LGG LIHC LUAD LUSC MESO OV PAAD PCPG PRAD READ SARC SKCM STAD TGCT THCA THYM UCEC UCS UVM
log10(HR)
1.0
0.5
0.0
−0.5
−1.0
B
Overall Survival
Low METTL5
High METTL5
Logrank p=0.0099
HR(high)=1.4
p(HR)=0.01
Overall Survival
Low RPUSD1
High RPUSD1
Logrank p=0.035
HR(high)=0.75
p(HR)=0.036
Overall Survival
Low DKC1
High DKC1
Logrank p=0.009
HR(high)=1.4
p(HR)=0.0095
C
Overall Survival
Low CTU1
High CTU1
Logrank p=0.00062
HR(high)=2.3
p(HR)=0.00087
Overall Survival
Low ALKBH5
High ALKBH5
Logrank p=0.044
HR(high)=0.62
p(HR)=0.047
Overall Survival
Low METTL14
High METTL14
Logrank p=0.04
HR(high)=1.6
p(HR)=0.042
Percent survival
Months

Figure S5

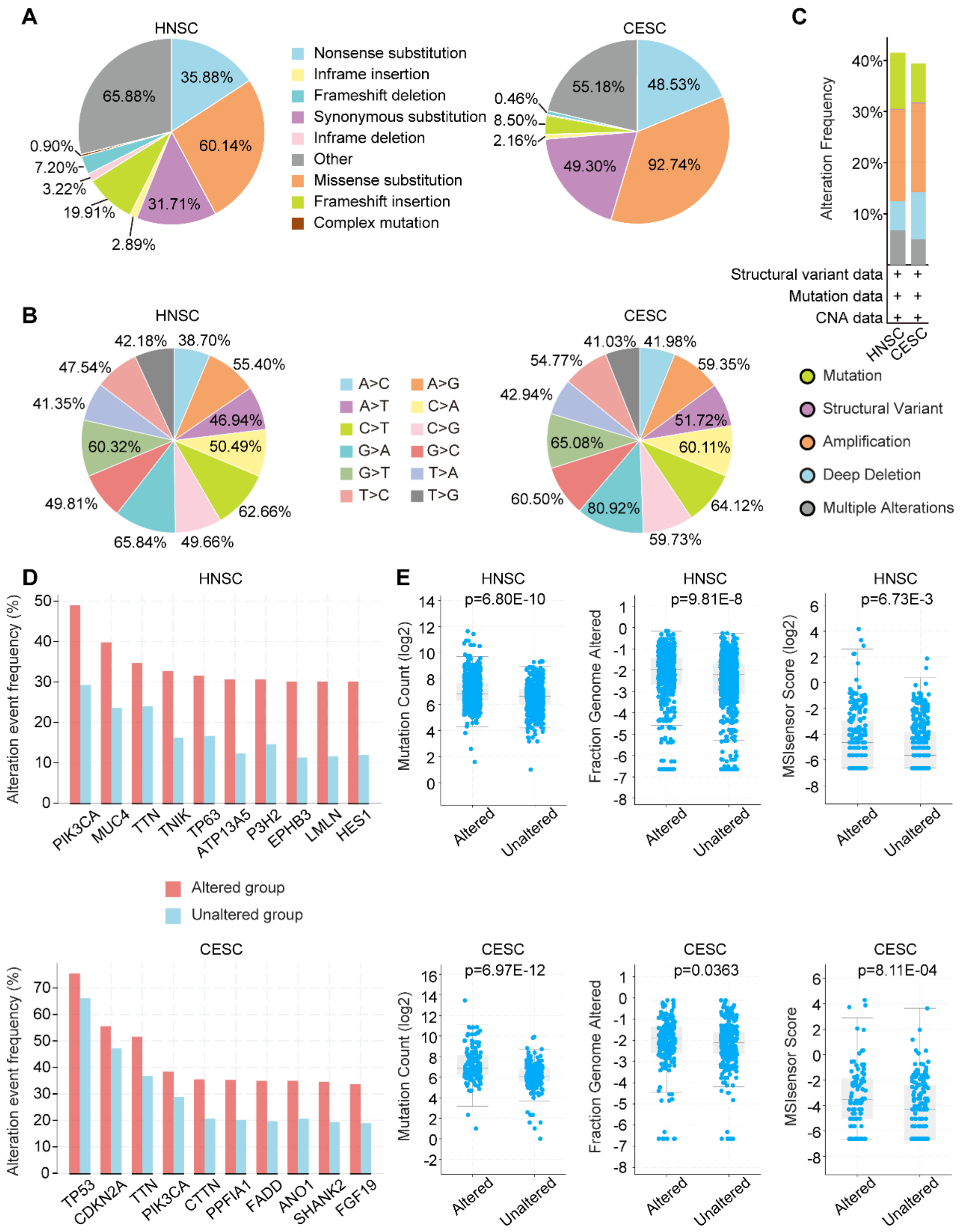

A
HNSC
CESC
Nonsense substitution
Inframe insertion
Frameshift deletion
Synonymous substitution
Inframe deletion
Other
Missense substitution
Frameshift insertion
Complex mutation
C
Alteration Frequency
Structural variant data
Mutation data
CNA data
B
A>C A>G A>T C>A C>T C>G G>A G>C G>T T>A T>C T>G
Mutation
Structural Variant
Amplification
Deep Deletion
Multiple Alterations
D
Alteration event frequency (%)
Altered group
Unaltered group
E
HNSC p=6.80E-10
HNSC p=9.81E-8
HNSC p=6.73E-3
CESC p=6.97E-12
CESC p=0.0363
CESC p=8.11E-04
Mutation Count (log2)
Fraction Genome Altered
MSIsensor Score (log2)
MSIsensor Score
Altered
Unaltered

Figure S6

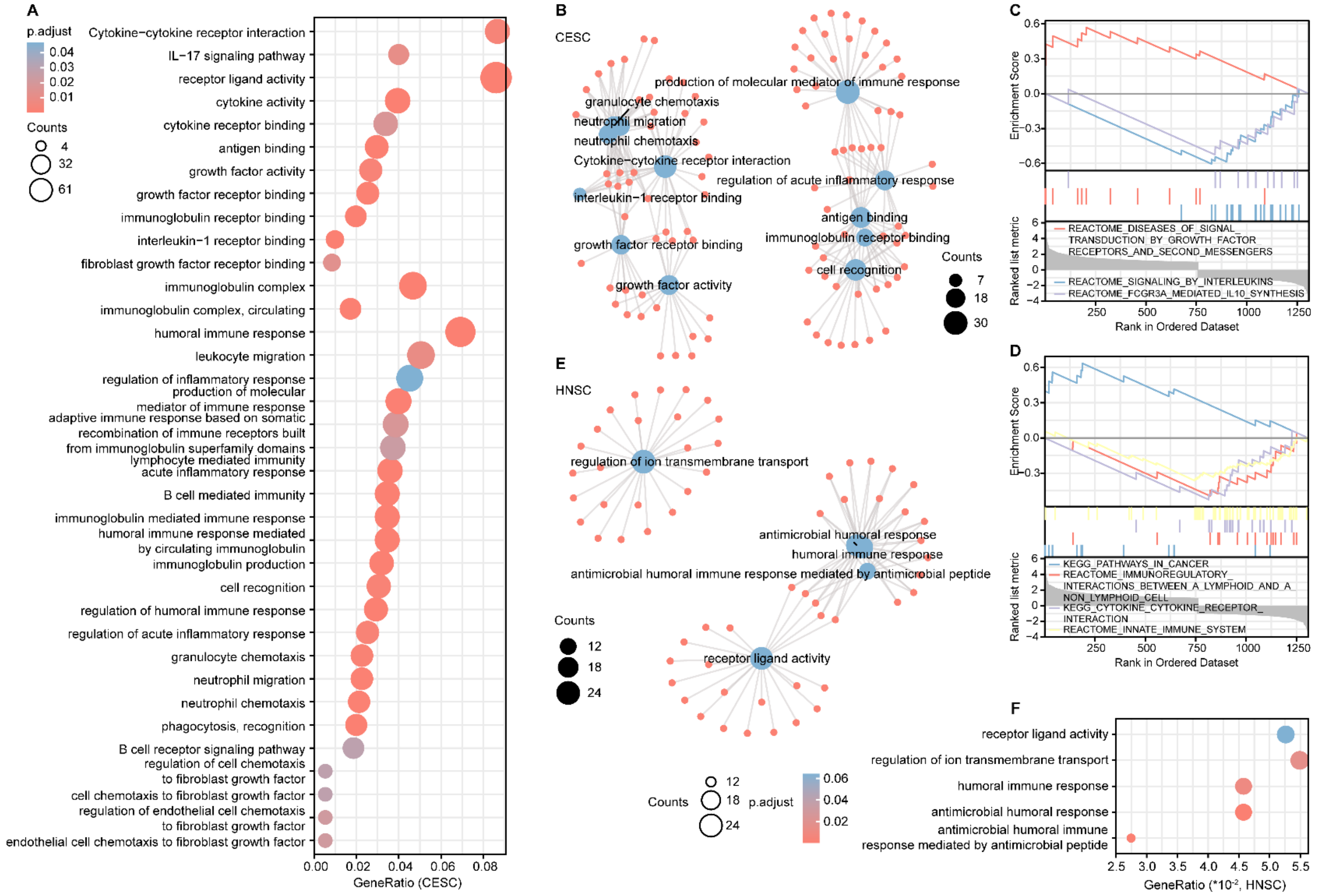
A
p.adjust
0.04
0.03
0.02
0.01
Counts
4
32
61
Cytokine−cytokine receptor interaction
IL−17 signaling pathway
receptor ligand activity
cytokine activity
cytokine receptor binding
antigen binding
growth factor activity
growth factor receptor binding
immunoglobulin receptor binding
interleukin−1 receptor binding
fibroblast growth factor receptor binding
immunoglobulin complex
immunoglobulin complex, circulating
humoral immune response
leukocyte migration
regulation of inflammatory response
production of molecular mediator of immune response
adaptive immune response based on somatic recombination of immune receptors built from immunoglobulin superfamily domains
lymphocyte mediated immunity
acute inflammatory response
B cell mediated immunity
immunoglobulin mediated immune response
humoral immune response mediated by circulating immunoglobulin
immunoglobulin production
cell recognition
regulation of humoral immune response
regulation of acute inflammatory response
granulocyte chemotaxis
neutrophil migration
neutrophil chemotaxis
phagocytosis, recognition
B cell receptor signaling pathway
regulation of cell chemotaxis to fibroblast growth factor
cell chemotaxis to fibroblast growth factor
regulation of endothelial cell chemotaxis to fibroblast growth factor
endothelial cell chemotaxis to fibroblast growth factor
0.00
0.02
0.04
0.06
0.08
GeneRatio (CESC)
B
CESC
production of molecular mediator of immune response
granulocyte chemotaxis
neutrophil migration
neutrophil chemotaxis
Cytokine−cytokine receptor interaction
regulation of acute inflammatory response
interleukin−1 receptor binding
antigen binding
immunoglobulin receptor binding
growth factor receptor binding
cell recognition
growth factor activity
Counts
7
18
30
C
Enrichment Score
0.6
0.3
0.0
−0.3
−0.6
Ranked list metric
6
4
2
0
−2
−4
REACTOME_DISEASES_OF_SIGNAL_TRANSDUCTION_BY_GROWTH_FACTOR_RECEPTORS_AND_SECOND_MESSENGERS
REACTOME_SIGNALING_BY_INTERLEUKINS
REACTOME_FCGR3A_MEDIATED_IL10_SYNTHESIS
250
500
750
1000
1250
Rank in Ordered Dataset
D
Enrichment Score
0.6
0.3
0.0
−0.3
Ranked list metric
6
4
2
0
−2
−4
KEGG_PATHWAYS_IN_CANCER
REACTOME_IMMUNOREGULATORY_INTERACTIONS_BETWEEN_A_LYMPHOID_AND_A_NON_LYMPHOID_CELL
KEGG_CYTOKINE_CYTOKINE_RECEPTOR_INTERACTION
REACTOME_INNATE_IMMUNE_SYSTEM
250
500
750
1000
1250
Rank in Ordered Dataset
E
HNSC
regulation of ion transmembrane transport
antimicrobial humoral response
humoral immune response
antimicrobial humoral immune response mediated by antimicrobial peptide
receptor ligand activity
Counts
12
18
24
Counts
12
18
24
p.adjust
0.06
0.04
0.02
F
receptor ligand activity
regulation of ion transmembrane transport
humoral immune response
antimicrobial humoral response
antimicrobial humoral immune response mediated by antimicrobial peptide
2.5
3.0
3.5
4.0
4.5
5.0
5.5
GeneRatio (*10⁻², HNSC)

Figure S7

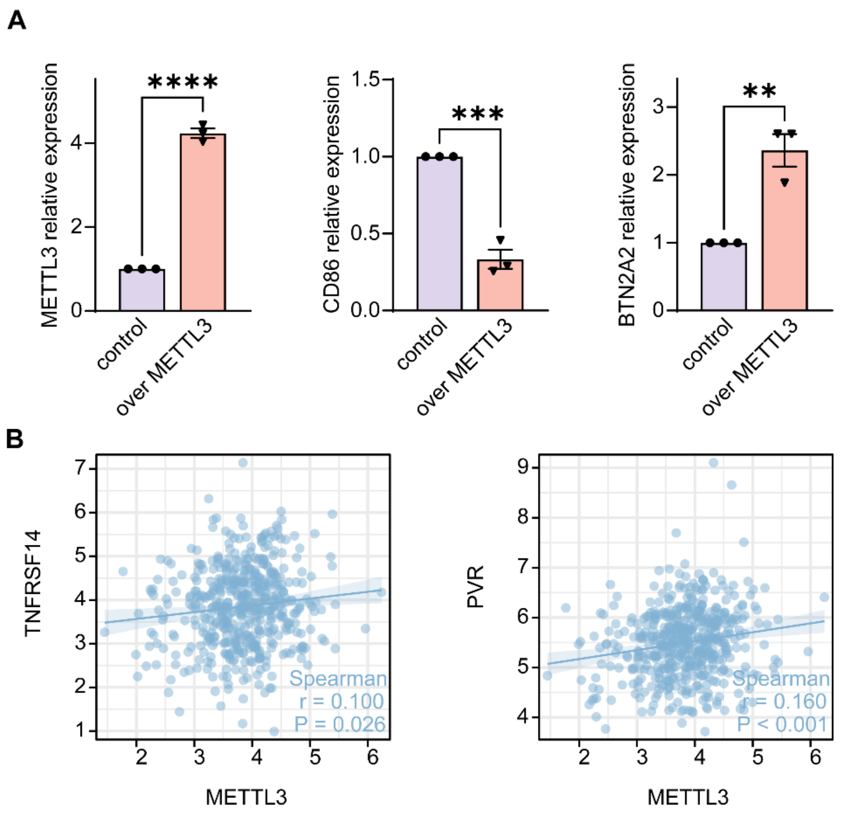
A
METTL3 relative expression
****
0
2
4
control
over METTL3
CD86 relative expression
***
0.0
0.5
1.0
1.5
control
over METTL3
BTN2A2 relative expression
**
0
1
2
3
control
over METTL3
B
TNFRSF14
METTL3
Spearman
r = 0.100
P = 0.026
PVR
METTL3
Spearman
r = 0.160
P < 0.001

Figure S8

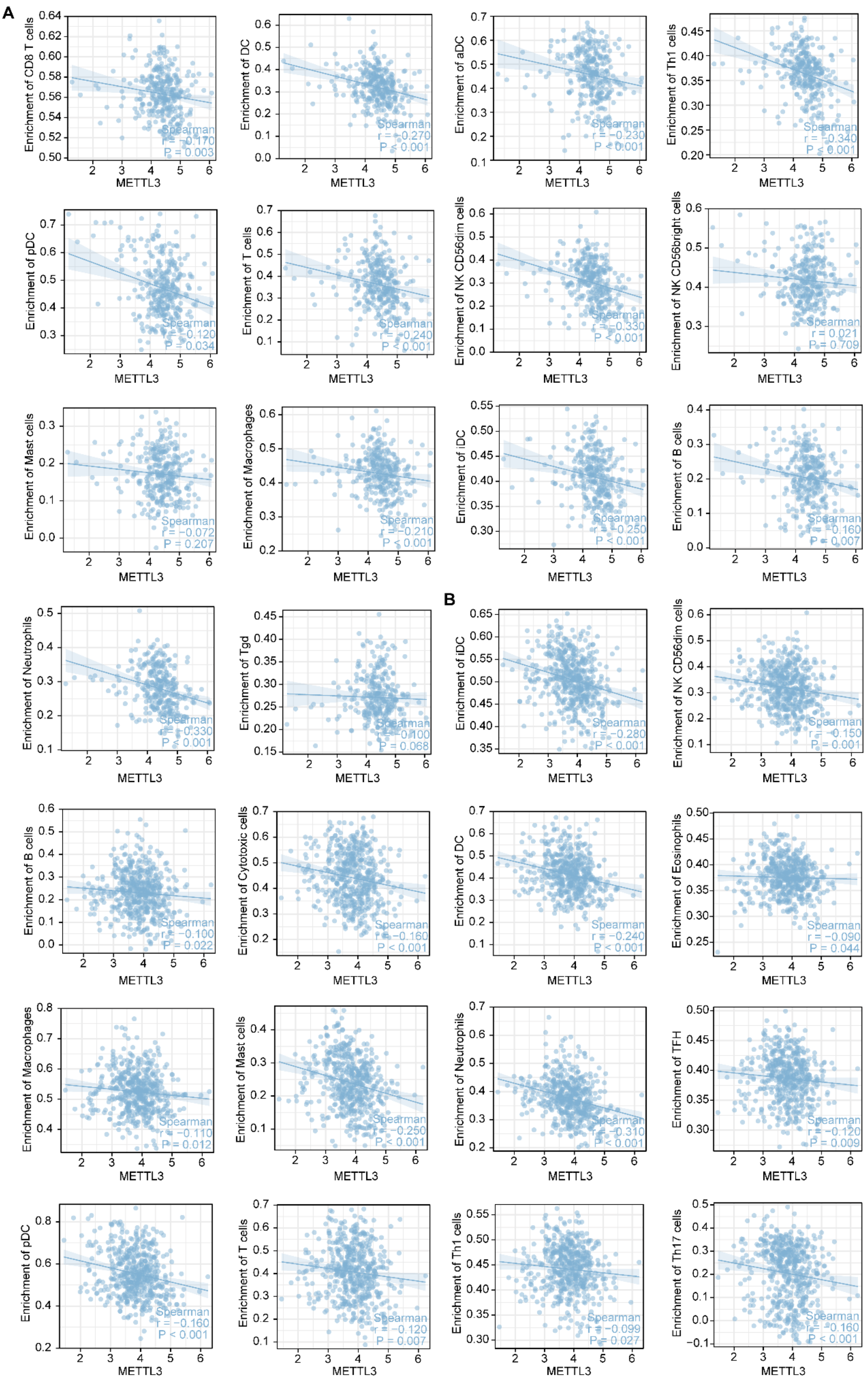
A
Enrichment of CD8 T cells
METTL3
Spearman
r = −0.170
P = 0.003
Enrichment of DC
METTL3
Spearman
r = −0.270
P < 0.001
Enrichment of aDC
METTL3
Spearman
r = −0.230
P < 0.001
Enrichment of Th1 cells
METTL3
Spearman
r = −0.340
P < 0.001
Enrichment of pDC
METTL3
Spearman
r = −0.120
P = 0.034
Enrichment of T cells
METTL3
Spearman
r = −0.240
P < 0.001
Enrichment of NK CD56dim cells
METTL3
Spearman
r = −0.330
P < 0.001
Enrichment of NK CD56bright cells
METTL3
Spearman
r = 0.021
P = 0.709
Enrichment of Mast cells
METTL3
Spearman
r = −0.072
P = 0.207
Enrichment of Macrophages
METTL3
Spearman
r = −0.210
P < 0.001
Enrichment of iDC
METTL3
Spearman
r = −0.250
P < 0.001
Enrichment of B cells
METTL3
Spearman
r = −0.160
P = 0.007
Enrichment of Neutrophils
METTL3
Spearman
r = −0.330
P < 0.001
Enrichment of Tgd
METTL3
Spearman
r = −0.100
P = 0.068
B
Enrichment of iDC
METTL3
Spearman
r = −0.280
P < 0.001
Enrichment of NK CD56dim cells
METTL3
Spearman
r = −0.150
P = 0.001
Enrichment of B cells
METTL3
Spearman
r = −0.100
P = 0.022
Enrichment of Cytotoxic cells
METTL3
Spearman
r = −0.160
P < 0.001
Enrichment of DC
METTL3
Spearman
r = −0.240
P < 0.001
Enrichment of Eosinophils
METTL3
Spearman
r = −0.090
P = 0.044
Enrichment of Macrophages
METTL3
Spearman
r = −0.110
P = 0.012
Enrichment of Mast cells
METTL3
Spearman
r = −0.250
P < 0.001
Enrichment of Neutrophils
METTL3
Spearman
r = −0.310
P < 0.001
Enrichment of TFH
METTL3
Spearman
r = −0.120
P = 0.009
Enrichment of pDC
METTL3
Spearman
r = −0.160
P < 0.001
Enrichment of T cells
METTL3
Spearman
r = −0.120
P = 0.007
Enrichment of Th1 cells
METTL3
Spearman
r = −0.099
P = 0.027
Enrichment of Th17 cells
METTL3
Spearman
r = −0.160
P < 0.001

Figure S9

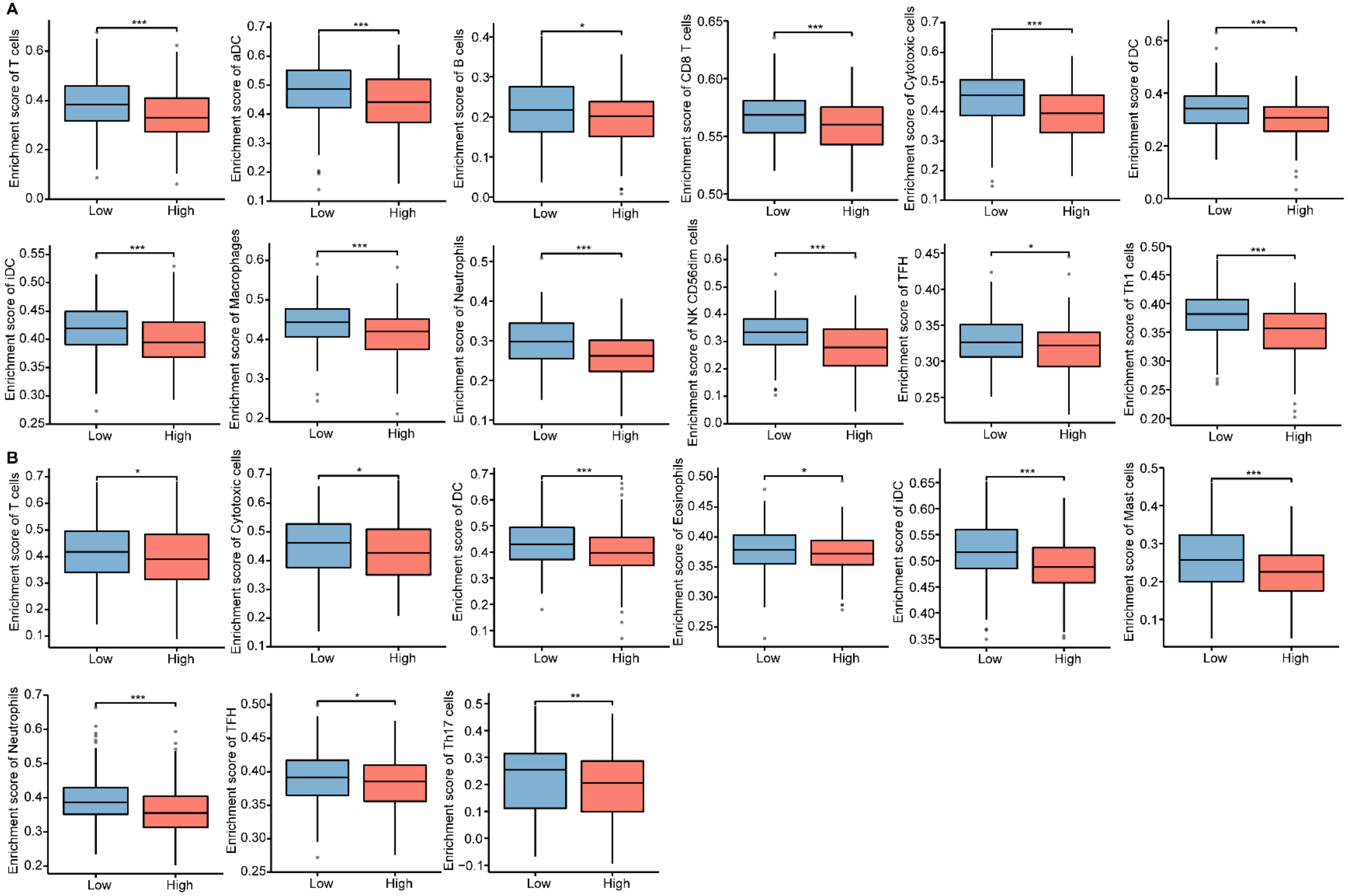
A
B
Enrichment score of T cells
Enrichment score of aDC
Enrichment score of B cells
Enrichment score of CD8 T cells
Enrichment score of Cytotoxic cells
Enrichment score of DC
Enrichment score of iDC
Enrichment score of Macrophages
Enrichment score of Neutrophils
Enrichment score of NK CD56dim cells
Enrichment score of TFH
Enrichment score of Th1 cells
Enrichment score of Eosinophils
Enrichment score of Mast cells
Enrichment score of Th17 cells
Low
High

Figure S10

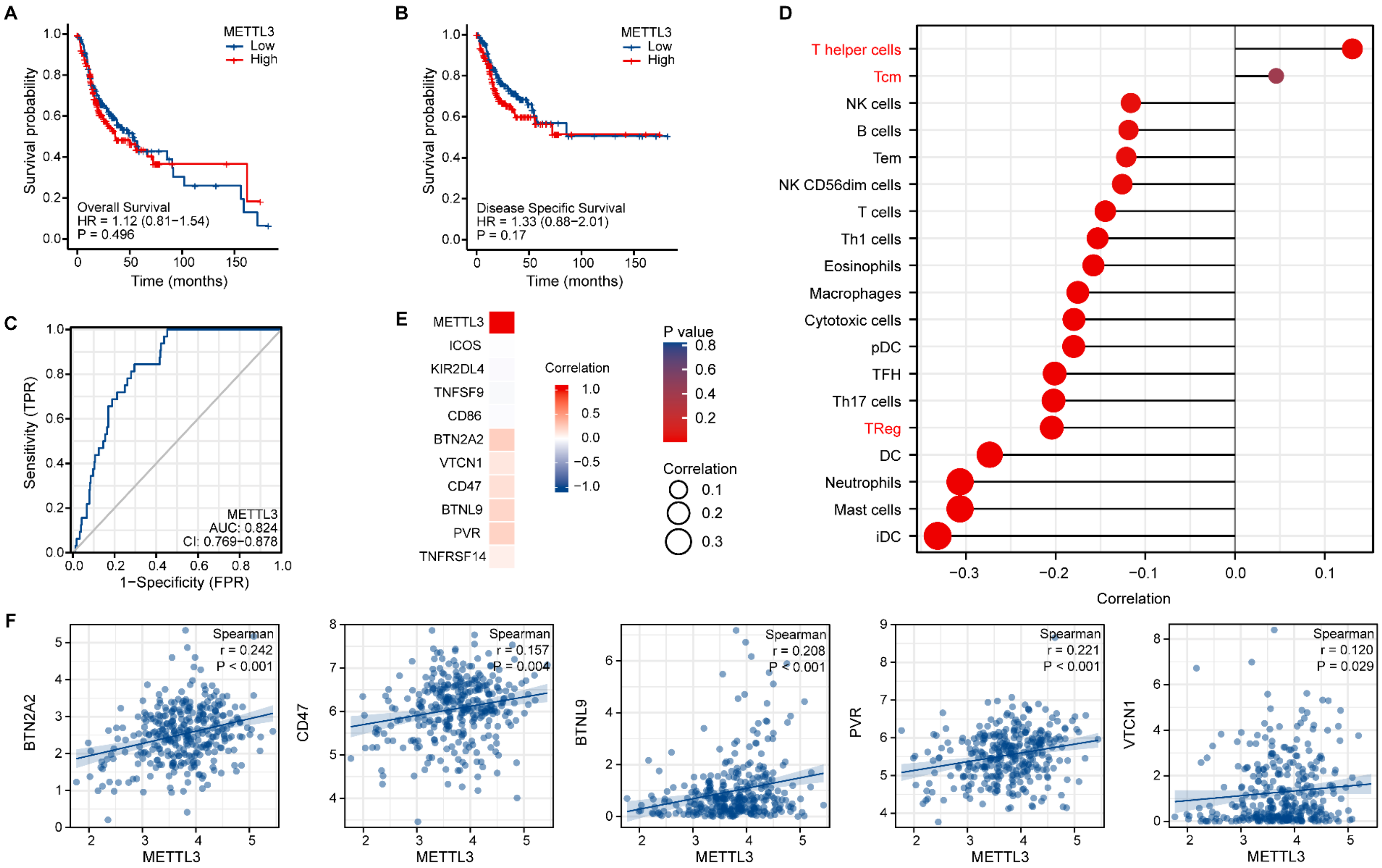

A
METTL3
Low
High
Survival probability
Overall Survival
HR = 1.12 (0.81−1.54)
P = 0.496
Time (months)
B
METTL3
Low
High
Survival probability
Disease Specific Survival
HR = 1.33 (0.88−2.01)
P = 0.17
Time (months)
C
Sensitivity (TPR)
1−Specificity (FPR)
METTL3
AUC: 0.824
CI: 0.769−0.878
E
METTL3
ICOS
KIR2DL4
TNFSF9
CD86
BTN2A2
VTCN1
CD47
BTNL9
PVR
TNFRSF14
Correlation
P value
Correlation
D
T helper cells
Tcm
NK cells
B cells
Tem
NK CD56dim cells
T cells
Th1 cells
Eosinophils
Macrophages
Cytotoxic cells
pDC
TFH
Th17 cells
TReg
DC
Neutrophils
Mast cells
iDC
Correlation
F
BTN2A2
Spearman
r = 0.242
P < 0.001
METTL3
CD47
Spearman
r = 0.157
P = 0.004
METTL3
BTNL9
Spearman
r = 0.208
P < 0.001
METTL3
PVR
Spearman
r = 0.221
P < 0.001
METTL3
VTCN1
Spearman
r = 0.120
P = 0.029
METTL3

Supplementary Table 1: Sequence of primers used in real-time PCR.

Primer	Sequence (5'to3')
TNFSF9-F	GGCTGGAGTCTACTATGTCTTCT
TNFSF9-R	ACCTCGGTGAAGGGAGTCC
BTN2A2-F	GGGCCAGCTAATCCCATCC
BTN2A2-R	GGTGATTCTTCCCCGGTACTC
CD274-F	GCTGCACTAATTGTCTATTGGGA
CD274-R	AATTCGCTTGTAGTCGGCACC
CD47-F	TCCGGTGGTATGGATGAGAAA
CD47-R	ACCAAGGCCAGTAGCATTCTT
PVR-F	GGACGGCAAGAATGTGACCT
PVR-R	GGTCGTGCTCCAATTATAGCCT
METTL3-F	AGATGGGGTAGAAAGCCTCCT
METTL3-R	TGGTCAGCATAGGTTACAAGAGT
GAPDH-F	ACAACTTTGGTATCGTGGAAGG
GAPDH-R	GCCATCACGCCACAGTTTC
KIR2DL4-F	TCCCTGTCCCTGAGCTCTAC
KIR2DL4-R	AAGGTCACGTTCTCTCCTGC
CD86-F	AGGCAACAATGAGCAGACCA
CD86-R	ACTATGGCTTGTTGGGTGGG
VTCN1-F	CTGCCTCTCAGCCCTTACCT
VTCN1-R	GCCTTCTGCTTTTGGCTTCTT
